# An empowerment programme to improve diet quality during pregnancy – the Power 4 a Healthy Pregnancy cluster randomised controlled trial

**DOI:** 10.1186/s12889-025-21344-z

**Published:** 2025-01-27

**Authors:** Renske M. van Lonkhuijzen, Jeanne H. M. de Vries, Elske Brouwer-Brolsma, Susanne Cremers, Janine P. M. Faessen, Edith J. M. Feskens, Annemarie Wagemakers

**Affiliations:** 1 Health and Society, Department of Social Sciences, Wageningen University & Research, Hollandseweg 1, Wageningen, 6706KN The Netherlands; 2 Division of Human Nutrition & Health, Wageningen University & Research, Stippeneng 4, Wageningen, 6708WE The Netherlands; 3 Bilthoven, The Netherlands

**Keywords:** Pregnancy, Diet quality, Empowerment, Cluster randomised controlled trial

## Abstract

**Background:**

A healthy diet during pregnancy is vital for the well-being of both mothers and babies. However, navigating dietary choices amidst the unique psychological and physiological changes of pregnancy can be challenging. Empowerment, defined as the ability to improve capacities, critically analyse situations, and take actions to improve them, can support pregnant women to make healthier choices. This intervention study assessed the effects of the ‘Power 4 a Healthy Pregnancy’ (P4HP) programme on diet quality and empowerment.

**Methods:**

In a nonblinded, two-arm, parallel cluster randomised controlled trial, the P4HP programme was implemented in 16 randomly allocated Dutch midwifery practices, recruiting 342 participants. Participants were assigned to either the intervention (*n* = 186) or the control group (*n* = 156). The P4HP programme offered four additional consultations during pregnancy to discuss nutrition with both a midwife and dietitian, using an empowerment approach. The effectiveness of the P4HP programme was evaluated using pre- and post-intervention questionnaires assessing diet quality, empowerment, quality of life, sense of coherence, and self-rated health. The data were analysed using linear mixed models with an intention-to-treat approach.

**Results:**

The P4HP programme was conducted from approximately week 11 to week 34 of pregnancy. The total diet quality score significantly improved during pregnancy in the intervention group compared to the control group (4.28; 95% CI: 1.00 to 7.56; *p* = 0.011), particularly driven by improvements in the scores for vitamin D, iodine, and fish. Although other components, including fruit, whole-grain foods, nuts, dairy foods, iodine, and fish showed greater average increases in diet quality scores within the intervention group, these differences were not significant. Women across all empowerment levels expressed uncertainty regarding their weight gain during pregnancy.

**Conclusion:**

The P4HP programme positively influenced the dietary habits of pregnant women through empowerment. The observed improvement in diet quality underscores the potential of the P4HP programme as an effective intervention during pregnancy. This study lays the foundation for future empowerment-based interventions in maternal health contexts.

**Trial registration:**

International Clinical Trial Registry Platform NL-OMON23191, date of registration: 19/05/2021.

**Supplementary Information:**

The online version contains supplementary material available at 10.1186/s12889-025-21344-z.

## Introduction

Maintaining a nutrient-rich diet during pregnancy is essential because of the health benefits to both expectant mothers and their unborn children. A healthy diet is universally pivotal but is particularly important during the gestational period [1, 2]. A set of specific dietary guidelines has been established for pregnant women by the Health Council of the Netherlands [3]. These guidelines aim to support the optimal development of the unborn baby. They recommend appropriate intake levels of essential nutrients, including vitamin D, folic acid, iodine, iron, and calcium. Additionally, the guidelines caution against consuming unsafe food groups, such as alcohol and liver products [3]. Such guidelines not only serve as a valuable resource for expectant mothers but also highlight the broader societal recognition of the critical role that maternal diet plays in the well-being of both present and future generations [4–6]. A suboptimal maternal diet not only correlates with adverse birth outcomes, such as premature birth and low birth weight, but also lays the foundation for unfavourable long-term health outcomes, including increased susceptibility to chronic diseases and obesity [5, 7].

Pregnancy, especially in nulliparous women, represents a unique opportunity during which individuals exhibit heightened receptivity to dietary improvements [8–12]. This critical transition period elevates nutritional awareness, creating a greater willingness among women to enhance their diet quality, motivated by the belief that it can positively influence their unborn babies’ well-being [8, 11, 13]. However, pregnancy poses challenges for women in terms of implementing and sustaining dietary changes [14]. Factors such as nausea, cravings, and established habits, as well as external challenges related to living costs and surroundings, contribute to this. Therefore, despite their motivation to enhance diet quality, pregnant women, especially those with low socioeconomic status, struggle to adhere to dietary recommendations [6, 15, 16]. A recent systematic review of food and nutrient intake among Dutch pregnant women revealed suboptimal intakes of fruits, vegetables, and fish while exceeding the recommendations for alcohol, sugary drinks, and salt [15]. Interestingly, the intake of other food groups or nutrients, such as protein and vitamin A, was found to be adequate [15].

Midwives serve as primary healthcare providers for pregnant women in the Netherlands, playing a crucial role that includes providing essential nutrition information and guidance during pregnancy [17–19]. This central role offers a valuable opportunity for promoting a healthy diet during pregnancy [17, 18, 20]. However, despite their responsibility to provide nutritional advice, midwives encounter structural barriers, such as time constraints, unsupportive health systems, and limited resources and training. These challenges contribute to suboptimal nutritional communication during antenatal care [18, 21–24].

Currently, only specific groups of Dutch women with pregnancy complications or weight-related concerns receive comprehensive nutritional guidance during pregnancy [18, 21, 25]. There is a strong need to strengthen the collaboration between dietitians and midwives, ensuring that nutrition becomes a routine and standard part of antenatal care [17, 18, 20]. The empowerment model suggests that by enhancing individuals' self-efficacy and decision-making skills, they are better equipped to adopt and sustain positive health behaviours [26]. Although the use of empowerment in nutrition-related programmes remains limited, it holds promise for health improvement [27–31]. Given these findings, integrating empowerment-based nutritional support could be valuable in promoting optimal maternal and foetal health among expectant Dutch mothers.

To address the lack of nutritional guidance, we developed the 'Power 4 a Healthy Pregnancy' (P4HP) programme aimed at improving prenatal diet quality, ultimately promoting healthier newborns and better health across generations. The P4HP programme was developed through preliminary research conducted by our team, including literature reviews, qualitative and quantitative studies [18, 20, 32–35], and a participatory co-design process with stakeholders (publication forthcoming). This thorough development phase ensured the programme met needs while remaining feasible within Dutch prenatal care settings. Motivational Interviewing (MI), a client-centred counselling approach, is used by participating dietitians and midwives during consultations. MI has shown promise across various health domains by recognizing individuals as experts in their own lives, emphasizing autonomy, and supporting personal choice in behaviour change [36, 37].

The objective of this study was to provide insight into the quantitative effectiveness of additional consultations within the P4HP programme primarily on diet quality and empowerment, and secondarily on self-rated health [SRH], quality of life [QOL], and sense of coherence [SOC]. As stated in the study protocol, we hypothesize that participation in the P4HP programme, in comparison to receiving standard prenatal care alone, would result in increased diet quality and empowerment scores among pregnant women [35]. By conducting this research, we aimed to contribute to the understanding of how empowerment-focused programmes can positively impact the diet quality and overall well-being of pregnant women, thereby influencing maternal health and pregnancy outcomes.

## Materials and methods

### Study design

The study design comprised a two-arm, non-blinded, parallel cluster randomised controlled trial (C-RCT) with a 1:1 allocation ratio. It was conducted across 16 midwifery practices in the Netherlands, from January 2022 to April 2023 [35]. Data were collected at two timepoints (T): baseline in early pregnancy (T0) and post-intervention in the third trimester (T1). Midwifery practices recruited pregnant women to participate in this study. During the study period, all the participants received standard prenatal care. Additionally, participants in the intervention group followed the P4HP programme along with standard prenatal care.

Before the start of the P4HP C-RCT study, a pilot study was conducted in two midwifery practices from October 2021 to January 2022. During this phase, practices 1 and 2 enrolled eight pregnant women (see Additional file 1 for characteristics of midwifery practices). We pretested the questionnaires, explored perceptions of the P4HP programme among pregnant women, midwives, and dietitians, and evaluated the practical and technical aspects of the implementation of the P4HP programme. The pilot phase progressed smoothly without significant issues, and no major modifications to the P4HP programme were deemed necessary. Consequently, we decided to include the participants and their data from the pilot phase in the C-RCT.

The CONSORT guidelines for cluster trials have been consulted [38]. The Medical Research Ethics Committee Utrecht (NedMec) approved this study on 21 September 2021 (protocol number 21–526/D). This study was registered in the WHO International Clinical Trial Registry Platform, before conducting the recruitment, on May 19th 2021 (NL-OMON23191) [39, 40].

### Recruitment of midwifery practices

Midwifery practices were recruited through Obstetric Collaborative Networks. These regional networks bring together various organizations involved in obstetric care, maternity care, and birth care to collaboratively establish pregnancy and birth care policies. We engaged local Solid Start (Kansrijke Start) coalitions as well as municipalities. These coalitions are Dutch government-initiated networks that promote collaboration between medical and social care professionals to ensure the best possible start in life for children. We employed snowball sampling for recruitment. We recruited midwifery practices from various regions across the Netherlands to enhance the applicability of this study to a broader population. An overview of the characteristics of participating midwifery practices is available in Additional file 1.

**Fig. 1:**
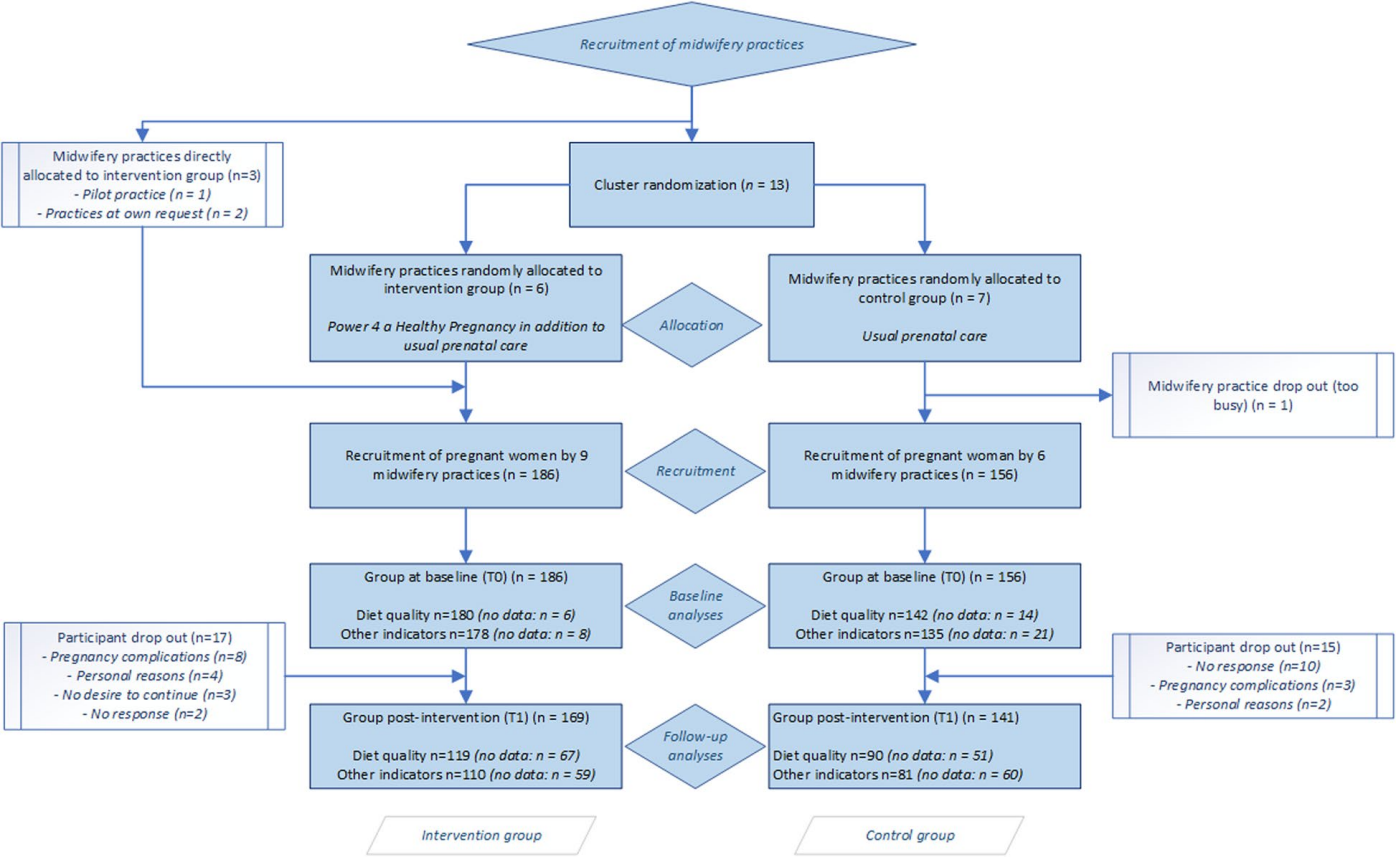
Flowchart illustrating participant recruitment and retainment, participation and drop-out rates in both the intervention and control groups of the P4HP study

We chose cluster randomisation for three key reasons. First, it prevented potential contamination between intervention and control groups within practices. Second, the P4HP programme required practice-level implementation of new workflows and collaborative relationships between midwives and dietitians. Third, the intervention's effectiveness depended on practice-wide organizational factors, making the practice the natural unit of randomization (Fig. 1). Of the two midwifery practices involved in the pilot study, only Practice 1 decided to continue its participation in the RCT. Based on their experience with implementing the P4HP programme during the pilot, we directly assigned Practice 1 to the intervention group. The subsequent 13 recruited practices (No. 3–15) were randomly allocated to either the intervention group (the P4HP programme in addition to usual prenatal care) or the control group (usual prenatal care). The randomization was performed by a computer-generated randomization scheme in Excel. Two additional midwifery practices (No. 16 and 17) were directly allocated to the intervention group, for several reasons. First, we reached our initial goal of 14 clusters (7 intervention and 7 control) [35]. Second, there was a notable imbalance in the participant recruitment distribution, resulting in a significantly larger control group. Third, practice no. 17 specifically requested participation in the intervention group to learn how to integrate nutrition into their practices, especially because of the high prevalence of overweight among their population. Unfortunately, one control midwifery practice (No. 15) discontinued study participation before participant recruitment started because of internal issues within the midwifery practice.

Midwifery practices with an existing collaboration with a dietitian were recruited together to participate in the P4HP programme (*n* = 2). For midwifery practices lacking current collaboration, we recruited dietitian practices within their region to participate in the P4HP programme. Subsequently, we facilitated introductory meetings between the dietitians and the eight midwifery practices in the intervention group. Approximately 40 midwives and 20 dietitians participated in the implementation of the P4HP programme. On average approximately 21 pregnant women (median: 26) were recruited from each midwifery practice, ranging from 3 to 32 (see Additional file 1 for characteristics of midwifery practices). Midwifery practices yielded complete datasets, containing four completed questionnaires (two at T0 and two at T1) for an average of approximately 12 pregnant women (median: 14). The percentage of complete datasets per midwifery practice ranged from 11% (1 of 9 women) to 86% (12 of 14 women).

### Participant recruitment

Eligible pregnant women were recruited using purposive sampling. During the predetermined inclusion period (January–October 2022), pregnant women attending any of the randomised participating midwifery practices were invited by their midwives to participate in the study. A total of 342 women were enrolled in the study; 186 were allocated to the intervention group and 156 to the control group (Fig. 1).

### Inclusion and exclusion criteria

To qualify, participants had to meet the following inclusion criteria: being in the first trimester of pregnancy, > 18 years of age, proficient in the Dutch language, and adhering to a Dutch diet pattern (characterised by a maximum of one hot meal per day). The exclusion criteria were: unwillingness to provide informed consent, severe chronic illnesses (e.g. cancer), and conditions potentially affecting diet quality. Eligible candidates were informed of the study by their midwives and written informed consent was obtained before inclusion. Women residing across a large part of the Netherlands had the opportunity to enrol via their midwifery practice.

### The P4HP programme

The P4HP programme was developed through an iterative process involving multiple stakeholders, e.g. midwives, dietitians, and pregnant women, who participated in expert meetings and stakeholder sessions to co-design the intervention, building on findings from our team's preliminary studies [18, 20, 32–35]. This participatory approach helped ensure the programme's practical feasibility and alignment with stakeholder needs. The subsequent pilot study in two midwifery practices allowed for final refinements based on implementation experience and stakeholder feedback before the main trial.

The P4HP programme distinguishes itself from conventional birth care by its non-invasive empowering approach, prioritising the improvement of diet quality during pregnancy. The P4HP programme consists of four additional opportunities during pregnancy for women to engage in discussions about nutrition: three consultations with their midwives, and one consultation with a dietitian. A summarised overview of the goals and activities of each consultation is presented in Table 1. The midwives and dietitians received comprehensive support to implement the P4HP programme. Professionals participated in multiple meetings with the research team, covering the detailed implementation of the P4HP programme in their practices. In addition, the detailed manual served as a comprehensive guide to support the delivery of the P4HP consultations (see Additional file 2 for the P4HP guide). By providing thorough preparation and ongoing support, we ensured the midwives and dietitians felt equipped to competently deliver the P4HP programme as intended. In addition, we assessed the process of implementing the P4HP programme and gathered feedback from the providers through a comprehensive process evaluation (Van Lonkhuijzen RM, Cremers S, De Vries JHM, et al: Midwives and dietitians’ perspectives on an empowerment programme to enhance diet quality in pregnant women: a mixed-method study, submitted).

**Table 1:** Summarized overview of the goals and activities in the four consultations of the P4HP programme, conducted between baseline (T0) and post-intervention (T1)

Time	Goal(s)	Activities
**1. Early nutritional information with the midwife (4–10 weeks of participants’ pregnancy)**		
10–15 min	- Gain insight into the perspectives of the women towards nutrition and create a sense of urgency for healthy nutrition during pregnancy - Identify aspects that go well regarding nutrition and define at least one achievable step the woman is willing to work on - Create a positive attitude towards nutritional changes - Understand the complexity of each woman’s situation regarding healthy nutrition	First exploratory consultation on nutrition. Optional use of visual aids to guide the consultation and write down action points.
**2. Appointment with the dietitian (~ 12 weeks of participants’ pregnancy)**		
30–45 min	- Support women in developing strategies to cope with individual challenges - Provide more in-depth nutritional information	Discuss challenges in healthy nutrition in-depth based on the women’s individual needs. Optional use of visual aids to guide the consultation and write down action points.
**3. Two reflection moments with the midwife (~ 22 and ~ 32 weeks of participants’ pregnancy)**		
2 × 10–15 min	- Raise awareness of the link between women’s actions and diet quality outcomes - Enhance the ability to set actionable and achievable goals that contribute to improved diet quality	Facilitate women’s reflection on their diet and their efforts during the previous weeks. Identify the next steps for improvement, if achievable.

The P4HP programme employs an empowering approach that prioritises individual needs, choices, and control. This approach allows midwives and dietitians to collaborate with pregnant women to identify the most effective strategies to their unique situations. As described by Tengland, empowerment is a process that offers individuals greater control over specific circumstances, thereby increasing their self-efficacy for those circumstances and tasks [41].

During P4HP consultations, health care professionals employ MI; a woman-centred and time-limited psychosocial intervention designed to identify and resolve behaviour disparities while increasing motivation for change [37]. Through open-ended questioning, MI empowers individuals by exploring their perceptions of change, including its meaning, importance and their capacity to achieve it [37, 42]. Research has demonstrated that MI is promising and can be more effective than standard education alone in improving pregnancy-related health behaviours, including fruit and vegetable consumption [36, 43]. For instance, a MI intervention with at-risk South African women led to significant reductions in alcohol-exposed pregnancy risk at both 3 and 12 months post-intervention [44]. In pregnancy care, MI is frequently used to influence gestational weight gain [45–48]. The approach is closely associated with empowerment principles, as both are rooted in self-determination theory, which suggests that individuals naturally strive to improve their wellbeing when they internalise the need for change [49]. While the combination of MI and empowerment approaches shows particular promise for supporting positive health behaviour changes, their joint application to prenatal nutrition remains understudied.

The P4HP programme combines the strengths of midwives and dietitians. The midwife, a highly trusted source of information for pregnant women, conducted three of the four P4HP consultations. Skilled in MI, the midwife reflected on nutrition together with the pregnant woman and helped to set and review goals. The dietitian, built on the midwife’s foundation, provided personalised nutritional guidance. Written consultation reports were exchanged between midwives and dietitians, allowing them to follow up on each other's consultations and ensure continuity of care. Emphasis was placed on repeated counselling and collaboration between midwives and dietitians to enhance the effectiveness of the programme [32–35, 50]. This way, enhancing the motivation of pregnant women and making behaviour changes became a shared endeavour rather than an individual responsibility [37, 51].

Both midwives and dietitians had the option to use a visual conversation tool to guide the consultations and document action points together with pregnant women (see Additional file 2 for the P4HP guide). Participating women received a magnet with the logo of the P4HP programme to have the option to stick their visual conversation tool on, for example, their fridge to have their goals in sight and to be reminded of them throughout the day.

Flexibility in implementing the P4HP programme was important for two main reasons: 1) to smoothly integrate the programme into the dynamic and varying daily procedures of the midwifery practice and 2) to provide freedom for professionals to tailor each consultation to the individual needs of the pregnant woman. Consequently, in two midwifery practices (No. 1 (post-pilot) and 16) dietitians conducted the first two P4HP consultations following explicit requests from the midwives. The early pregnancy period was intensive for midwives, making it inconvenient for them to accommodate the first P4HP consultation within their routines. Additionally, dietitians expressed a preference for multiple consultations with pregnant women.

Midwives and dietitians were fully reimbursed for the time invested while participating women had no additional costs compared with standard birth care. Initially, the P4HP programme was planned to be implemented in either individual or group settings; however, due to the COVID-19 pandemic, group consultations via CenteringPregnancy were disrupted. Therefore, it was decided to conduct the P4HP programme exclusively on an individual basis.

### Data collection

Data collection began when participants provided written informed consent. Two questionnaires were developed for this study; an adapted version of the Eetscore questionnaire and a questionnaire on sociodemographics, empowerment, and health (see Additional file 3 for the questionnaires). The Eetscore questionnaire, which is scored using the Dutch Healthy Diet index for pregnant women (DHD-P), is a web-based screening tool designed to assess diet quality. It typically takes 10–15 min to complete [52–55]. The DHD-P was designed to align with the dietary recommendations for pregnant women set by The Health Council of the Netherlands (HCN) and the Netherlands Nutrition Centre, making it particularly suitable for our study population and research aim [35, 56, 57]. The DHD-P evaluates 21 food and nutrient components through 48 questions with sub-questions [57]. Each component was scored on a scale of 0 (non-adherence) to 10 (complete adherence), resulting in a maximum of 210 points. However, because supplementation with folic acid is not recommended in the final stage of pregnancy, we excluded the folic acid component, resulting in a maximum total diet quality score of 200. The scoring has been described in detail elsewhere [57] and is summarised in Table 2 including the cut-off and threshold values.

**Table 2:** Cut-off and threshold values for diet quality scores

	**Component**^**a**^	**Dutch dietary guidelines**	**Minimum score (= 0 points)**	**Maximum score (= 10 points)**
1	Vegetables	At least 200 g per day	No intake	200 g or more per day
2	Fruit	At least 200 g per day	No intake	200 g or more per day
3	Whole-grain products *(Both components contribute 50% of the score)*	At least 90 g of whole grain	No intake	90 g or more whole grains per day
		Replace refined cereals with wholegrain products	No intake of whole grains OR ratio of wholegrain/refined products ≤ 0.7	No refined products OR ratio wholegrain/refined products ≥ 11
4	Legumes	Eat legumes weekly	No intake	10 g or more per day
5	Nuts	At least 15 g of unsalted nuts per day	No intake	15 g or more per day
6	Dairy products	Consume about 3 to 4 portions of dairy per day (450–600 g) including milk and yoghurt, with a maximum of 40 g of cheese	No intake OR > 750 g per day	450–600 g per day
7	Fish *(50% score based on fatty fish once a week and 50% of the score is based on lean fish once a week.)*	Twice a week fish, of which one time a week fatty fish and one time a week lean fish. Avoid fish that are not advised for pregnant women	No intake	30 g or more per day
8	Caffeine	No more than 200 mg of caffeine (calculated from coffee and tea consumption)	More than 200 mg of caffeine per day	Less than 200 mg caffeine per day
9	Fat and oils	Replace butter, hard margarines and cooking fats by soft margarines, liquid cooking fats and vegetable oils	No consumption of soft margarines, liquid cooking fats and vegetable oils OR ratio of liquid cooking fats to solid cooking fats = < 0.6	No consumption of butter, hard margarines and cooking fats OR Ratio of liquid cooking fats to solid cooking fats = > 13
10	Coffee	Replace unfiltered coffee by filtered coffee	Any consumption of unfiltered coffee	Consumption of only filtered coffee OR no coffee consumption
11	Red meat	Minimize consumption, maximum of 300 g per week	100 g or more per day	45 g or less per day
12	Processed meat	Minimize consumption, maximum of 200 g per week	50 g or more per day	No intake
13	Sugar-containing beverages	Minimize consumption	1 glass (250 g) or more per day	No intake
14	Alcohol	No consumption	Any amount of consumption	No intake
15	Salt^b^	Limit consumption to 6 g/day (2.4 g/day of sodium)	3042 mg or more per day	Less than 2028 mg per day
16	Unhealthy choices	Limit consumption of unhealthy day and week choices to ≤ 3 week choices per week	More than seven choices per week	Three or less choices per week
17	Vitamin D	10 µg per day supplementation	No supplementation OR 100 mcg or more per day	10 µg per day
18	Vitamin A	750 mcg RAE per day and not more than 3000 mcg RAE per day	0 mcg RAE OR 3000 mcg RAE or more per day	750 mcg RAE per day
19	Soy	Limit soy consumption to no more than 4 portions of soy milk/yoghurt per day and no more than 2 portions of soy products (tempeh, tofu, etc.) per week. Unless you do not consume soy milk/yoghurt, then there is no limitation on soy products such as tempeh and tofu	More than 4 portions per day of soy milk/yoghurt and/or more than 2 portions per week of soy products (if any soy milk/yoghurt is consumed)	Consumption below 4 portions per week of soy milk/yoghurt and below 2 portions of soy products per week (if any soy milk/yoghurt is consumed)
20	Iodine	Consume sufficient products containing iodine to obtain 200 mcg per day	No consumption of fish, dairy and bread	2 times a week fish, 3–4 portions dairy and 5 slices of bread per day

^a^As supplementation of folic acid is not recommended in the final stage of pregnancy, we excluded the folic acid component from our analyses resulting in a maximum total diet quality score of 200

^b^All Eetscore items together do not cover the entire daily diet. Therefore, it is necessary to apply a correction for the cut-off values of sodium. To establish the cut-off values, the Health Council recommendation (maximum 6 g salt = 2400 mg sodium) was multiplied by the MOM1 value for sodium (84.51%): 2400 mg * 0.8451 = 2028 mg. To arrive at the upper limit, a maximum of 9 g of salt (= 3600 mg of sodium) per day was assumed to be multiplied by the MOM1 value for sodium, amounting to 3042 mg

The questionnaire on sociodemographics, empowerment, and health was administered using the Qualtrics platform. Sociodemographic data, including age, living situation, ethnicity, educational level, personal and household income, and body weight and height, were collected. The subsequent section of the questionnaire consisted of the Pregnancy-Related Empowerment Scale (PRES), a tool used to assess the level of empowerment during pregnancy [58]. This scale is rooted in the concept of health-related empowerment and contains 16 questions on women’s health-related empowerment during pregnancy divided into four subscales: provider connectedness, skilful decision-making, peer connectedness, and gaining voice [58]. Pregnant women's level of empowerment increases as the scale score increases, with 16 being the lowest possible score and 64 being the highest. Additionally, the health outcomes of SRH, QOL, and SOC were assessed. Participants rated their SRH on a five-point scale, ranging from ‘excellent’ to ‘poor’. QOL was evaluated using the global QoL scale, with respondents rating their satisfaction on a visual analogue scale ranging from 0 (worst) to 100 (best) [59]. SOC was assessed using the three-item SOC questionnaire (SOC-3), which assesses the comprehensibility, manageability, and meaningfulness of daily life experiences [60]. Participants responded to each item as “usually”, “sometimes”, or “no”.

On average, the questionnaires at T0 were completed at approximately week 11 of pregnancy (range 5–21 weeks of pregnancy), and at T1, they were completed at approximately 34 weeks of pregnancy (range 30–39 weeks of pregnancy). Both the control and intervention groups were asked to complete the Eetscore questionnaire to collect information about diet quality along with a questionnaire on sociodemographic information, empowerment, and health [35]. Participants were first e-mailed a request to complete the questionnaires. In the case of non-completion, participants were repeatedly reminded to complete the questionnaires via e-mail and phone. When we failed to contact the pregnant woman for multiple weeks, we contacted her midwifery practice to check her situation.

### Power calculation

A total sample size of 350 participants was required to detect a small to medium effect size of 0.4 [61] with a power of 80%, an alpha level of 0.05, and an assumed intra-cluster correlation coefficient of 2%. Both the intervention and control arms were intended to comprise 7 clusters of 25 participants each. Ultimately, a slightly smaller number of 342 participants were enrolled in the study, but this discrepancy was compensated for by including more midwifery practices (clusters) than originally planned, thereby enhancing the statistical power [35]. A total of 32 participants dropped out of the study: 15 from the control group and 17 from the intervention group, resulting in a dropout rate of 9.4% (Fig. 1). Follow-up measurements on diet quality were available for 209 participants (61.1% of initially recruited participants), 119 in the intervention, and 90 in the control practices.

### Data analysis

Descriptive data analyses were performed using SPSS Statistics (version 28.0) and are presented (as mean ± SD or n (%) for the intervention and control groups separately. Independent t-tests (continuous variables) and independent Chi-square Test of Independence (categorical variables) were used to compare the descriptive characteristics between the study groups. In addition, we stratified the population into two groups of lower and higher-than-average diet quality at T1. We assessed the differences for these groups for all DHD-P subscores to identify which diet quality components contribute most to the differences in diet quality between groups with below and above-average total diet quality scores at T1 (see Additional file 4).

The effects of the intervention on endline values of primary and secondary outcomes were analysed using Stata release 18 [62]. Mixed Models with intervention vs. control as fixed effect, midwifery practice (cluster) as a random factor [63] and baseline values (T0) as covariates, as recommended [64], and robust variance estimators were used. We analysed the data according to the intention-to-treat principle [65]. Additionally, a Last Observation Carried Forward (LOCF) analysis was executed for total diet quality to account for dropout rates, ensuring the inclusion of all participants in the analysis, and providing a more precise effect estimate. Missing data at T1 were imputed using each participant's T0 data. All models were adjusted for BMI and household income. Normally distributed outcome variables such as DHD-P total score were analysed using the Stata procedure ‘mixed’, DHD-P subscores with the procedure ‘metobit’ (mixed tobit regression with lower limit 0 and upper limit 10), and DHD-P subscores with categorical distributions, PRES, SOC, and SRH were analysed with the procedure ‘meologit’ (mixed ordinal regression). *P* values < 0.05 were considered to indicate statistical significance.

## Results

### Descriptive characteristics

On average, the 342 participants were 31 years old (Table 3). Nearly half of the women experienced their first pregnancy. Most women were Dutch, highly educated, and employed during pregnancy, with no differences between the intervention and control groups. Yet, women in the intervention group had a significantly higher BMI (25.9 ± 5.6 vs. 24.7 ± 4.7 kg/m^2^, P < 0.05) and household income (32% vs. 17% reporting ≥ € 5.001,-) compared to the control group. Baseline scores of the two primary outcomes, diet quality and empowerment, were comparable between the two groups.

**Table 3:** Descriptive characteristics of the 342 P4HP C-RCT-study participants at baseline

		**Intervention**	**Control**	***P*** **value**
**Age (y) (mean ± SD)** *(n* = *287)*		31.0 ± 4.3	31.3 ± 3.9	.50
**BMI (kg/m**^**2**^**) (mean ± SD)** *(n* = *282)*		25.9 ± 5.6	24.7 ± 4.7	< .05
**BMI groups (kg/m2) (n (%))** *(n* = *282)*	< 18.5	3 (1.9)	4 (3.2)	.17
	18.5–25	80 (51.0)	75 (60.0)	
	25–30	44 (28.0)	33 (26.4)	
	30 +	30 (19.1)	13 (10.4)	
**Parity (n (%))** *(n* = *312)*	Primiparous	87 (50.0)	58 (42.0)	.16
	Multiparous	87 (50.0)	80 (58.0)	
**Migration background (n (%))** *(woman and/or at least one of parents born outside of the Netherlands) (n* = *317)*		27 (15.2)	18 (12.9)	.57
**Paid employment during pregnancy (n (%))** *(n* = *314)*		167 (93.8)	122 (89.7)	.18
**Education (n (%))** *(n* = *314)*	Secondary education	10 (5.6)	2 (1.5)	.19
	Secondary vocational education	56 (31.5)	46 (33.8)	
	Higher professional education	67 (37.6)	59 (43.4)	
	University education	45 (25.3)	29 (21.3)	
**Monthly net household income (n (%))** *(n* = *314)*	€ 0.- to € 2.500,-	12 (6.7)	11 (8.1)	.03
	€ 2.501,- to € 5.000,-	89 (50.0)	82 (60.3)	
	€ 5.001,- and more	57 (32.0)	23 (16.9)	
	I do not know/I do not want to answer this question	20 (11.2)	20 (14.7)	
**Total diet quality score (mean ± SD)** *(n* = *322)*		134.9 ± 17.1	135.9 ± 17.3	.60
**Total empowerment score** *(n* = *313)*		58.0 ± 5.7	58.7 ± 3.8	.22

### Diet quality

**Fig. 2:**
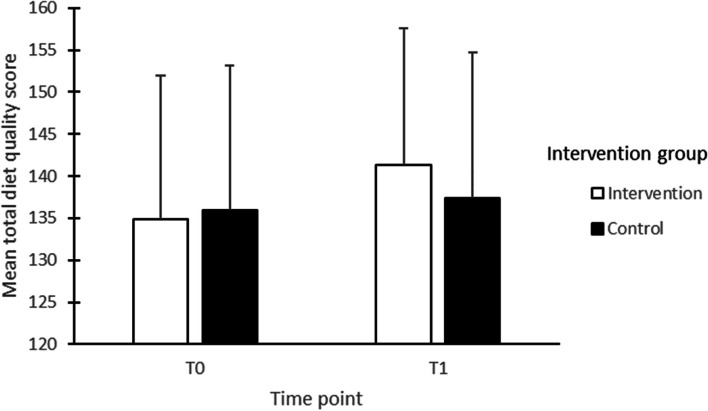
The average of total diet quality scores of 342 pregnant women at baseline (T0; on average 11 weeks of pregnancy) and post-intervention (T1; on average 34 weeks of pregnancy) implementing the P4HP programme

Figure 2 presents the intervention effects on diet quality, with both groups showing improvements from the first to third trimesters. This increase was significantly higher in the intervention group than in the control group (4.28; 95% CI: 1.00 to 7.56; *p* = 0.011). Applying the LOCF method, to handle missing data, did not substantially alter the results, but did provide a more precise estimate, showing a slightly lower group-difference (2.75; 95% CI: 0.76 to 4.72; *p* = 0.007).

Based on the DHD-P subscores of the total diet quality score, the effect found for the total diet score was particularly driven by a higher intake of fish (0.55, 95% CI: 0.07 to 1.03, *p* = 0.025), and iodine (0.40, 95% CI: 0.20 to 0.61; p < = 0.0001), and smaller decrease in vitamin D intake (0.48; 95% CI: 0.02 to 0.95; *p* = 0.043) in the intervention compared to the control group. Although components such as fruit, whole-grain products, nuts, dairy products, processed meat, and sugar-containing beverages showed greater average increases in diet quality scores in the intervention group than in the control group, the differences were not statistically significant on a stand-alone basis (Table 4). Nevertheless, stratifying the total population into two subsets, those with under and above-average total diet quality scores at T1, showed the potential to improve the diet quality of nuts (3.9), legumes (3.8), vegetables (2.7), fruit, (2.0), fat and oils (2.0), and processed meat (2.0). Large differences between the subsets demonstrate the potential to improve the diet quality in this population (see Additional file 4).

**Table 4:** Total diet quality score and 20 components scores for 342 pregnant women, before and after the Power 4 a Healthy Pregnancy program

DHD Components	Time	Intervention		Control		Difference between groups	
		(Mean (SD)	Median [IQR 25, 75]	Mean (SD)	Median [IQR 25, 75]	Estimate (95% CI)	*P*
Total score^a^ (range 0–200)	T0	134.9 (17.1)	135.0 [121.3, 146.0]	135.9 (17.3)	135.0 [125.0, 146.3]	4.28 (1.00 to 7.56)	.011
	T1	141.3 (16.3)	141.0 [131.0, 153.0]	137.4 (17.3)	145.5 [127.5, 153.0]		
1 Vegetables	T0	6.4 (2.9)	6.7 [4.2, 9.4]	6.3 (3.1)	6.3 [4.3, 9.7]	0.13 (−0.80 to 1.07)	.779
	T1	6.5 (2.8)	6.8 [4.2, 9.1]	6.1 (3.2)	6.4 [3.5, 9.7]		
2 Fruit	T0	7.6 (2.5)	8.8 [6.9, 10.0]	7.8 (2.5)	8.8 [6.9, 10.0]	0.30 (−0.75 to 1.34)	.578
	T1	8.1 (2.4)	8.8 [6.9, 10.0]	7.7 (2.5)	8.8 [6.9, 8.8]		
3 Whole-grain products	T0	7.1 (2.8)	7.2 [5.1, 10.0]	7.1 (2.7)	6.9 [5.5, 10.0]	0.47 (−0.36 to 1.30)	.264
	T1	7.6 (2.6)	7.8 [6.0, 10.0]	7.6 (2.4)	7.9 [5.8, 10.0]		
- Gram	T0	4.5 (1.1)	5.0 [5.0, 5.0]	4.6 (1.0)	5.0 [5.0, 5.0]		
	T1	4.6 (1.0)	5.0 [5.0, 5.0]	4.8 (0.8)	5.0 [5.0, 5.0]		
- Ratio	T0	2.6 (2.1)	2.3 [0.5, 5.0]	2.6 (2.1)	2.0 [0.5, 5.0]		
	T1	3.0 (2.0)	3.1 [1.0, 5.0]	2.8 (2.0)	2.9 [0.9, 5.0]		
4 Legumes	T0	5.3 (4.5)	6.5 [0.0, 10.0]	5.5 (4.4)	6.5 [0.0, 10.0]	1.23 (−1.73 to 4.18)	.416
	T1	6.2 (4.3)	8.7 [1.1, 10.0]	6.5 (4.2)	8.7 [2.2, 10.0]		
5 Nuts	T0	4.1 (3.5)	2.9 [1.0, 6.8]	3.7 (3.4)	2.9 [1.0, 5.8]	1.01 (−0.35 to 2.38)	.146
	T1	4.8 (3.7)	2.9 [1.0, 8.7]	4.0 (3.7)	2.9 [1.0, 6.8]		
6 Dairy products	T0	4.1 (2.8)	3.5 [1.7, 6.1]	4.4 (3.0)	3.9 [1.6, 6.5]	0.58 (−0.13 to 1.28)	.108
	T1	5.1 (2.8)	4.4 [3.2, 7.0]	4.6 (2.7)	4.0 [2.5, 6.8]		
7 Fish	T0	4.0 (2.80)	3.9 [2.0, 5.7]	3.7 (3.1)	3.1 [1.3, 5.6]	0.55 (0.07 to 1.03)	.025
	T1	4.6 (2.86)	4.9 [2.0, 6.8]	3.8 (2.9)	3.2 [1.3, 5.4]		
- Lean	T0	2.2 (1.68)	1.4 [0.7, 2.9]	2.0 (1.8)	1.4 [0.7, 4.0]		
	T1	2.5 (1.73)	2.9 [1.1, 4.3]	2.1 (1.6)	1.4 [0.7, 2.9]		
- Fatty	T0	1.9 (1.7)	1.3 [0.6, 3.8]	1.7 (1.7)	1.3 [0.0, 2.5]		
	T1	2.1 (1.7)	1.3 [0.6, 3.8]	1.7 (1.6)	1.3 [0.0, 2.5]		
8 Caffeine	T0	7.7 (4.2)	10.0 [10.0, 10.0]	8.4 (3.7)	10.0 [10.0, 10.0]	−0.24 (−0.69 to 0.21)	.305
	T1	7.4 (4.4)	10.0 [0.0, 10.0]	8.6 (3.5)	10.0 [10.0, 10.0]		
9 Fat and oils	T0	6.4 (4.5)	10.0 [0.4, 10.0]	6.5 (4.4)	10.0 [1.2, 10.0]	−0.32 (−3.13 to 2.48)	.820
	T1	6.3 (4.6)	10.0 [0.3, 10.0]	6.7 (4.4)	10.0 [1.1, 10.0]		
10 Coffee	T0	8.1 (2.5)	10.0 [5.0, 10.0]	8.4 (2.6)	10.0 [5.0, 10.0]	−0.15 (−0.61 to 0.32)	.530
	T1	7.9 (2.6)	10.0 [5.0, 10.0]	8.2 (2.5)	10.0 [5.0, 10.0]		
11 Red meat	T0	9.5 (1.7)	10.0 [10.0, 10.0]	9.9 (0.5)	10.0 [10.0, 10.0]	0.24 (−1.44 to 1.92)	.783
	T1	9.8 (1.1)	10.0 [10.0, 10.0]	9.9 (0.6)	10.0 [10.0, 10.0]		
12 Processed meat	T0	5.4 (3.4)	5.5 [3.1, 8.3]	5.4 (3.5)	5.5 [2.9, 8.5]	−0.02 (−1.03 to 0.99)	.964
	T1	6.0 (3.3)	7.1 [4.1, 8.5]	5.6 (3.4)	6.4 [2.5, 8.5]		
13 Sugar-containing beverages	T0	6.9 (3.5)	8.3 [4.0, 10.0]	6.3 (3.6)	7.8 [3.4, 9.5]	−0.26 (−0.75 to 0.24)	.305
	T1	7.5 (3.1)	9.1 [5.7, 10.0]	6.8 (3.5)	8.3 [5.0, 9.6]		
14 Alcohol	T0	9.4 (2.4)	10.0 [10.0, 10.0]	9.9 (1.2)	10.0 [10.0, 10.0]	1.29 (−0.09 to 2.67)	.066
	T1	10.0 (0.0)	10.0 [10.0, 10.0]	10.00 (0.0)	10.0 [10.0, 10.0]		
15 Salt	T0	8.5 (1.8)	9.0 [8.5, 9.5]	8.7 (1.5)	9.0 [8.5, 9.5]	−0.19 (−0.65 to 0.27)	.420
	T1	8.7 (1.8)	9.0 [8.5, 9.5]	8.9 (1.4)	9.5 [8.5, 9.5]		
16 Unhealthy choices	T0	3.0 (3.7)	0.9 [0.0, 5.5]	3.3 (3.9)	1.3 [0.0, 6.6]	0.38 (−1.90 to 2.67)	.744
	T1	2.9 (3.8)	0.0 [0.0, 6.3]	2.8 (3.9)	0.0 [0.0, 5.9]		
17 Vitamin D	T0	8.4 (2.9)	10.0 [8.9, 10.0]	7.6 (3.7)	10.0 [5.0, 10.0]	0.48 (0.02 to 0.95)	.043
	T1	8.3 (2.5)	9.4 [7.8, 10.0]	6.8 (3.8)	9.4 [5.0, 10.0]		
18 Vitamin A	T0	8.6 (1.7)	10.0 [6.7, 10.0]	8.7 (1.7)	10.0 [6.7, 10.0]	1.19 (−034 to 0.71)	.486
	T1	8.8 (1.7)	10.0 [6.7, 10.0]	8.6 (1.9)	10.0 [6.7, 10.0]		
19 Soy	T0	9.7 (1.7)	10.0 [10.0, 10.0]	9.8 (1.4)	10.0 [10.0, 10.0]	−0.13 (−2.47 to 2.22)	.916
	T1	9.8 (1.3)	10.0 [10.0, 10.0]	9.9 (1.1)	10.0 [10.0, 10.0]		
20 Iodine	T0	4.5 (1.7)	4.4 [3.4, 5.4]	4.5 (1.8)	4.6 [3.3, 5.7]	0.40 (0.20 to 0.61)	.000
	T1	5.2 (1.8)	5.2 [3.9, 6.4]	4.7 (1.6)	4.7 [3.6, 5.8]		

^a^Folic acid was excluded because supplementation of folic acid is not recommended in the final stage of pregnancy

Two midwifery practices in the intervention group accounted for a large increase in diet quality (see Additional file 1), as the diet quality score of women in practice no. 11 increased on average by 12.0 points and of women from practice no. 16 on average by 11.2 points. The range of change in the average total diet quality score among the participating women was −11.3 to 14.0 for intervention practices and −5.2 to 8.4 for control practices.

### Empowerment

Table 5 displays the results for the total empowerment score along with the scores for the 16 individual statements. The average total empowerment score showed a small increase in both the intervention group (T0: mean: 58.0 (SD: 5.7) T1: mean: 59.5 (SD: 4.6)) and the control group (T0: mean: 58.7 (SD: 3.8) T1: mean: 59.5 (SD: 4.4)), with a difference of 0.178 (95% CI: −1.52 to 1.88; *p* = 0.824).

**Table 5:** Total empowerment and 16 component scores for 342 pregnant women, before and after the Power 4 a Healthy Pregnancy program

	Time	Intervention		Control		Difference between groups	
		(Mean (SD)	Median [IQR 25, 75]	Mean (SD)	Median [IQR 25, 75]	Estimate (95% CI)	*P*
**Empowerment – total score**	T0	58.0 (5.7)	59.0 [57.0, 61.0]	58.7 (3.8)	59.0 [57.0, 61.0]	0.08 (−0.44 to 0.59)	.771
	T1	59.5 (4.6)	60.0 [58.0, 62.0]	59.5 (4.4)	60.0 [58.5, 62.0]		
**Provider connectedness**							
1. I can ask my health care provider about my pregnancy.	T0	3.9 (0.5)	4.0 [4.0, 4.0]	3.9 (0.4)	4.0 [4.0, 4.0]	0.24 (−0.80 to 1.27)	.656
	T1	3.9 (0.4)	4.0 [4.0, 4.0]	3.9 (0.5)	4.0 [4.0, 4.0]		
2. I have enough time with my health care provider to discuss my pregnancy.	T0	3.8 (0.5)	4.0 [4.0, 4.0]	3.8 (0.5)	4.0 [4.0, 4.0]	0.32 (−1.24 to 1.88)	.688
	T1	3.8 (0.5)	4.0 [4.0, 4.0]	3.8 (0.5)	4.0 [4.0, 4.0]		
3. My health care provider listens to me.	T0	3.9 (0.4)	4.0 [4.0, 4.0]	3.9 (0.4)	4.0 [4.0, 4.0]	0.17 (−1.96 to 2.30)	.875
	T1	3.9 (0.4)	4.0 [4.0, 4.0]	3.9 (0.5)	4.0 [4.0, 4.0]		
4. My health care provider respects me.	T0	3.9 (0.4)	4.0 [4.0, 4.0]	3.9 (0.4)	4.0 [4.0, 4.0]	0.10 (−1.62 to 1.81)	.913
	T1	3.9 (0.4)	4.0 [4.0, 4.0]	3.9 (0.4)	4.0 [4.0, 4.0]		
5. I expect my health care provider to respect my decisions about my pregnancy.	T0	3.8 (0.6)	4.0 [4.0, 4.0]	3.9 (0.4)	4.0 [4.0, 4.0]	0.19 (−1.27 to 1.65)	.797
	T1	3.9 (0.4)	4.0 [4.0, 4.0]	3.9 (0.4)	4.0 [4.0, 4.0]		
6. My health care provider respects my decision, even if it is different than her/his recommendation.	T0	3.7 (0.6)	4.0 [3.0, 4.0]	3.7 (0.6)	4.0 [4.0, 4.0]	.21 (−0.42 to 0.85)	.505
	T1	3.7 (0.5)	4.0 [3.0, 4.0]	3.7 (0.6)	4.0 [3.0, 4.0]		
**Skilful decision-making**							
7. I take responsibility for the decisions I make about my pregnancy like eating healthy food.	T0	3.9 (0.4)	4.0 [4.0, 4.0]	3.9 (0.3)	4.0 [4.0, 4.0]	−0.04 (−1.21 to 1.13)	.947
	T1	3.9 (0.4)	4.0 [4.0, 4.0]	3.9 (0.4)	4.0 [4.0, 4.0]		
8. I can tell when I have made a good health choice.	T0	3.6 (0.5)	4.0 [3.0, 4.0]	3.7 (0.5)	4.0 [3.0, 4.0]	−0.33 (−0.88 to 0.23)	.254
	T1	3.7 (0.6)	4.0 [3.0, 4.0]	3.8 (0.5)	4.0 [4.0, 4.0]		
9. Since I began prenatal care, I have been making more decisions about my health.	T0	3.1 (0.8)	3.0 [3.0, 4.0]	3.1 (0.9)	3.0 [3.0, 4.0]	−0.02 (−0.43 to 0.40)	.938
	T1	3.2 (0.8)	3.0 [3.0, 4.0]	3.2 (0.8)	3.0 [3.0, 4.0]		
**Peer connectedness**							
10. Women need to share experiences with other women when they are pregnant.	T0	3.4 (0.6)	3.0 [3.0, 4.0]	3.3 (0.8)	3.0 [3.0, 4.0]	−0.27 (−0.81 to 0.27)	.332
	T1	3.4 (0.6)	3.0 [3.0, 4.0]	3.4 (0.6)	3.0 [3.0, 4.0]		
11. I share my feelings and experiences with other women.	T0	3.2 (0.7)	3.0 [3.0, 4.0]	3.1 (0.9)	3.0 [3.0, 4.0]	−0.17 (−0.71 to 0.38)	.552
	T1	3.3 (0.7)	3.0 [3.0, 4.0]	3.2 (0.8)	3.0 [3.0, 4.0]		
**Gaining voice**							
12. I know if I am gaining the right amount of weight during my pregnancy.	T0	2.9 (0.8)	3.0 [2.0, 3.0]	3.1 (0.8)	3.0 [3.0, 4.0]	0.69 (0.14 to 1.36)	.045
	T1	3.5 (0.7)	4.0 [3.0, 4.0]	3.4 (0.7)	4.0 [3.0, 4.0]		
13. I have a right to ask questions when I don’t understand something about my pregnancy.	T0	3.9 (0.4)	4.0 [4.0, 4.0]	4.0 (0.2)	4.0 [4.0, 4.0]	0.09 (−1.13 to 1.32)	.882
	T1	3.9 (0.3)	4.0 [4.0, 4.0]	4.0 (0.2)	4.0 [4.0, 4.0]		
14. I am able to change things in my life that are not healthy for me.	T0	3.6 (0.6)	4.0 [3.0, 4.0]	3.7 (0.5)	4.0 [3.0, 4.0]	−0.10 (−0.85 to 0.65)	.803
	T1	3.7 (0.5)	4.0 [3.0, 4.0]	3.8 (0.4)	4.0 [4.0, 4.0]		
15. I am doing what I can to have a healthy baby.	T0	3.8 (0.5)	4.0 [4.0, 4.0]	3.9 (0.4)	4.0 [4.0, 4.0]	−0.45 (−1.04 to 0.15)	.142
	T1	3.8 (0.4)	4.0 [4.0, 4.0]	3.9 (0.3)	4.0 [4.0, 4.0]		
16. If something is going wrong in my pregnancy, I know who to talk to.	T0	3.8 (0.5)	4.0 [4.0, 4.0]	3.8 (0.4)	4.0 [4.0, 4.0]	0.07 (−0.72 to 0.86)	.867
	T1	3.9 (0.4)	4.0 [4.0, 4.0]	3.9 (0.3)	4.0 [4.0, 4.0]		

Women scored high at baseline for most PRES statements, with a mean of 3.8 or 3.9 out of 4.0. However, the scores for PRES statement 12, ‘I know if I am gaining the right amount of weight during my pregnancy’, significantly differed from the other statements. At baseline, the scores for statement 12 were comparable between the intervention group (T0 mean: 2.9, SD: 0.8) and the control group (T0 mean: 3.1, SD: 0.8). The mean for statement 12 increased more in the intervention group (T1 mean: 3.5, SD: 0.7) than in the control group (T1 mean: 3.4, SD: 0.7), with a difference of 0.69 (95% CI: 0.14 to 1.36; *p* = 0.045).

### Secondary outcomes

All secondary outcomes, including QOL, SRH and SOC, showed similar patterns in both groups and remained relatively stable between T0 and T1 and showed no statistically significant differences (Table 6). No adverse events were reported during the trial.

**Table 6:** Self-rated health, quality of life and sense of coherence scores for 342 pregnant women, before and after the Power 4 a Healthy Pregnancy program

	Time	Intervention		Control		Difference between groups	
		(Mean (SD)	Median [IQR 25, 75]	Mean (SD)	Median [IQR 25, 75]	Estimate (95% CI)	*P*
**Self-Rated Health**	T0	4.0 (0.7)	4.0 [4.0, 4.0]	4.2 (0.6)	4.0 [4.0, 5.0]	−0.22 (−0.66 to 0.22)	.325
	T1	4.2 (0.7)	4.0 [4.0, 5.0]	4.3 (0.7)	4.0 [4.0, 5.0]		
**Quality of Life**	T0	7.8 (1.5)	8.0 [7.0, 9.0]	8.1 (1.2)	8.0 [7.0, 9.0]	0.15 (−0.36 to 0.66)	.559
	T1	8.0 (1.4)	8.0 [7.0, 9.0]	8.0 (1.2)	8.0 [7.5, 9.0]		
**Sense of Coherence – Total score** *weak (scores 6–9), intermediate (scores 4–5), and strong (score 3)*	T0	4.2 (1.0)	4.0 [3.0, 5.0]	4.1 (1.1)	4.0 [3.0, 5.0]	0.31 (−0.30 to 0.92)	.323
	T1	4.0 (1.0)	4.0 [3.0, 5.0]	(0.9)	4.0 [3.0, 4.0]		
- Do you usually see a solution to problems and difficulties that other people find hopeless? (manageability)	T0	1.5 (0.5)	1.0 [1.0, 2.0]	1.5 (0.6)	1.0 [1.0, 2.0]	0.06 (−0.54 to 0.66)	.841
	T1	1.4 (0.5)	1.0 [1.0, 2.0]	1.4 (0.5)	1.0 [1.0, 2.0]		
- Do you usually feel that your daily life is a source of personal satisfaction? (meaningfulness)	T0	1.4 (0.5)	1.0 [1.0, 2.0]	1.2 (0.5)	1.0 [1.0, 1.0]	0.59 (−0.03 to 1.21)	.062
	T1	1.3 (0.5)	1.0 [1.0, 1.0]	1.1 (0.3)	1.0 [1.0, 1.0]		
- Do you usually feel that the things that happen to you in your daily life are hard to understand? (comprehensibility)	T0	1.3 (0.5)	1.0 [1.0, 2.0]	1.3 (0.5)	1.0 [1.0, 2.0]	0.44 (−0.25 to 1.12)	.215
	T1	1.3 (0.5)	1.0 [1.0, 2.0]	1.2 (0.5)	1.0 [1.0, 1.0]		

## Discussion

The present study aimed to quantitatively evaluate the impact of the P4HP programme on diet quality and empowerment among 342 pregnant women through a C-RCT. Our study was among the first to employ a collaborative midwife-dietitian empowerment programme to improve the diet quality of pregnant women. A key finding of this study was the improvement in total diet quality scores within the intervention group compared to the control group, with a difference of + 4.3 units. Among the individual components, fish, iodine, and vitamin D intake exhibited significant differences between the intervention and control groups. These outcomes underscore the promising potential of the P4HP programme to positively influence the dietary habits of pregnant women through the use of empowerment as a means. Given the absence of prior research with a similar focus, this research not only contributes to existing knowledge but also highlights the potential for implementing empowerment-based interventions within maternal health contexts.

We found a significant improvement in diet quality, but the clinical relevance of this improvement remains unclear. There is no consistent evidence regarding the extent to which diet quality directly impacts the health of child-bearing women and their offspring [66]. However, there is broad consensus that pregnant women need to maintain a balance in body weight and blood pressure, with a nutrient-dense diet significantly contributing to achieving this balance.

This study demonstrated potential food components where the diet quality in this population can be improved. Certain food groups are more prevalent in Dutch dietary patterns than others. For example, adherence to fruit consumption guidelines is generally high, while legumes and nuts are less common in Dutch diets [67]. The greatest differences in diet quality components between groups with below- and above-average total diet quality scores at T1 were evident for nuts, legumes, vegetables, fruit, fat and oils, and processed meat, suggesting the potential for change in these areas. It may be advisable to prioritise these components in nutritional communication.

There is considerable diversity in programmes aimed at improving nutrition behaviour among pregnant women. A systematic review of systematic reviews revealed that behaviour-change interventions have generally been successful in increasing fruit and vegetable consumption and reducing carbohydrate intake during pregnancy [68]. For example, the educational workshop-based programme 'The Healthy Start to Pregnancy' showed improvements in health behaviours such as increased fruit intake [69]. In contrast, the women-held record ‘The Pregnancy Pocketbook’ showed no effect on fruit and vegetable consumption [70]. The Be Healthy in Pregnancy study, which provided bi-weekly nutrition counselling, led to improved diet quality and protein intake, but did not significantly impact gestational weight gain [71]. Additionally, several studies focusing on managing gestational weight gain have failed to find significant intervention effects [72, 73].

The P4HP programme significantly improved iodine intake in the intervention group. While there are indications that iodine intake is declining across the Dutch population, little is known about iodine intake among pregnant Dutch women [15, 74]. A large-scale study is ongoing to assess iodine intake among pregnant women in the Netherlands, contributing to the knowledge base in this research area [15, 75]. If this study finds a generally inadequate intake of iodine among pregnant Dutch women, the P4HP programme could be highly relevant, offering the advantage of addressing nutrition comprehensively.

While the P4HP programme incorporated empowerment principles, the similar empowerment scores between groups suggest that the improvements in diet quality may have stemmed from other programme elements, such as the additional repeated nutritional guidance or the collaborative care involving midwives and dietitians. Using the PRES, which evaluates both external and internal attributes of empowerment and their relationship to health outcomes [76], we found high baseline empowerment scores across most domains, with participants consistently scoring near the maximum of 4, leaving limited room for improvement. However, a notable exception emerged regarding weight gain awareness (statement 12), where the intervention group showed significant improvement. The uncertainty about gestational weight gain was not unique to our study population. Similar patterns have been observed among diverse populations, including low-income pregnant African American and Hispanic women in the United States [58].

Research indicates that pregnancy-related weight concerns can influence patterns of gestational weight gain [77]. For example, a Norwegian cohort study involving 36,000 pregnant women demonstrated that increased worry about weight gain was associated with higher actual weight gain [78]. The significant improvement in statement 12 ('I know if I am gaining the right amount of weight during my pregnancy') among intervention participants suggests that the P4HP programme effectively addressed this common concern. This improvement occurred despite participants' generally high baseline empowerment levels, indicating that even highly empowered women benefit from structured support regarding gestational weight management. These findings support previous research emphasising the importance of incorporating interprofessional weight gain guidance into prenatal care programmes, regardless of women's baseline empowerment levels [79, 80].

The evaluation of the intervention's impact extended beyond diet quality and empowerment by including SOC, QOL, and SRH. This comprehensive evaluation aimed to provide a holistic understanding of the programme's effects on participants' overall well-being. The short duration of this study and the generally high scores at baseline are possibly attributable to the relatively stable QOL, SOC, and SRH in this study. A high SOC for pregnant women is related to better well-being, reduced anxiety and a more favourable predisposition to depression [81, 82]. According to Antonovsky’s theory, SOC is a stable disposition of personality, and pregnancy and delivery are not considered radical life events that significantly affect the degree of a woman’s SOC [83, 84]. Indeed, no significant effect was detected in the SOC scores of this study, which is similar to the findings of research among 177 pregnant women in Sweden [81]. However, it is important to note that women whose pregnancies ended or who experienced serious complications dropped out of this study (*n* = 11), which may have influenced this outcome. In our study, QOL for women in the intervention group improved on average by + 0.2, while for women in the control group, it decreased on average by −0.1. Lagadec et al. systematically reviewed the quality of life of pregnant women, showing a significant decrease in physical QOL throughout the trimesters [85]. Similarly, Boutib et al. reported that QOL in the 9th month of pregnancy was lower than that in the 3rd month [86]. Therefore, achieving stability or a slight increase in QOL over such a short time, as observed in the intervention group, represents a positive outcome. While SRH related to pregnancy has been investigated, little is known about changes in SRH during pregnancy [87].

Given that empowerment was a key component of the P4HP programme, quantitative measures alone may not fully capture all of its beneficial effects. To gain a more comprehensive understanding, we conducted a complementary process evaluation using surveys and in-depth interviews with pregnant women, midwives, and dietitians. These surveys and interviews aimed to explore individual experiences, programme impacts, and implementation experiences (Van Lonkhuijzen RM, Cremers S, De Vries JHM, et al: Midwives and dietitians’ perspectives on an empowerment programme to enhance diet quality in pregnant women: a mixed-method study, submitted; Van Lonkhuijzen RM, Prins S, van Loghem F, et al: Pregnant women’s experiences with an empowerment programme to improve diet quality, submitted). This evaluation demonstrated considerable variability in the goals women set for healthier dietary intake, resulting in diverse outcomes across different dietary components.

### Study strengths and limitations

This trial has several strengths. Firstly, through this randomised trial, we are pioneering the execution of a programme specifically designed to empower pregnant women to make healthier dietary choices. Secondly, a strength of this pragmatic trial lies in its comparison of a relevant alternative to current practice in real-world settings, thereby providing generalizable results that can be directly applied in routine practice settings.

In addition, several limitations should be considered. The first concerns the timing of baseline measurements. The T0 measurements, taken at approximately week 11, may have missed the earlier pregnancy phase when women's motivation to adhere to dietary guidelines is typically strongest [88]. Earlier measurements were not feasible due to standard Dutch birth care procedures, which include an initial midwife consultation between weeks 8–10, a first ultrasound between weeks 10–12, and a second consultation around weeks 14–16. As a result, study enrolment and the informed consent processes had to align with these standard care timepoints.

The second limitation concerns the characteristics of the study population. While the PRES has been validated previously, our study population showed high baseline empowerment scores, potentially creating a ceiling effect that may have limited our ability to detect changes related to the intervention. This finding raises important considerations for future research. First, it suggests that Dutch pregnant women, particularly those who voluntarily participate in nutrition-focused interventions, may already feel highly empowered in their healthcare interactions. Second, it indicates that future interventions might benefit from more sensitive measurement tools or alternative approaches when targeting highly empowered populations. Rather than viewing this as a methodological failure, we consider it an important insight for the design of future maternal health interventions. Tailored approaches may be needed for populations with varying levels of baseline empowerment, and recruitment strategies may need modification to better engage less empowered populations. This observation aligns with our finding regarding uncertainty around weight gain across all empowerment levels, suggesting that even highly empowered women benefit from specific forms of support during pregnancy.

Third, a notable challenge in the study was the relatively high number of missing values at T1, which reduced the power of the analysis. Throughout the study, researchers reached out to participating women repeatedly through various means, encouraging them to complete the questionnaires. Despite these efforts, many participants did not complete all of the questionnaires, resulting in differences in the percentage of complete datasets across midwifery practices. One possible explanation for this trend is that women in the early stages of their pregnancy may have been more engaged and motivated to monitor their lifestyle and diet. While in pregnancy, their focus may have shifted to the final preparations for delivery and the arrival of their baby, resulting in decreased participation. Another factor affecting the retrieval of follow-up data is the extent to which the women were reminded by the midwifery practice. To address this, we used the LOCF analysis, which allowed for the inclusion of all participants in the final analysis, ensuring the generalisability of the findings, and showing the robustness of the primary results. However, it is important to note that the LOCF analysis, assumes that the diet quality at T0 is a valid estimate for missing values at T1, which may not always be accurate.

Fourth, baseline BMI was higher in the intervention group, potentially reflecting greater scope for dietary improvements, as suggested by the slightly lower baseline diet quality score. Though all models were adjusted for BMI, BMI data should be interpreted cautiously due to potential reporting errors and its association with dietary misreporting [89], which may affect intervention outcomes. Measuring the self-reported BMI of participating pregnant women proved challenging due to variations in gestational age when completing the questionnaire [90]. It is worth noting that weight gain during the first trimester is typically minimal, which may partially mitigate this limitation [91].

Fifth, a methodological consideration was that three midwifery practices were directly assigned to the intervention group based on their interest in nutrition support. While this non-random allocation might suggest potential bias, these practices showed varied outcomes (−11.3 to + 11.2 points in diet quality), indicating that practice assignment alone did not determine intervention effectiveness. Rather, these variations highlight how implementation success likely depends on multiple factors, including provider engagement and practice culture.

Finally, the DHD-P has two limitations. First, participants were required to adhere to a traditional Dutch diet pattern in order to be assessed. This may have excluded individuals from migrant backgrounds who do not typically follow this dietary pattern. As a result, the findings may have limited applicability to more diverse populations with different cultural food preferences and eating habits. Future research should explore the effectiveness of motivational interviewing for dietary behaviour change in more heterogeneous samples that better reflect the diversity of the general population. A second limitation is the use of scoring truncation, where each component is restricted to a score ranging from 0 to 10. This constraint limits the index’s capacity to capture all variations in dietary habits. Also, no weighting was applied to the nutrient and food components. By weighting, more importance could be given to components that are more relevant for pregnancy outcomes [92].

### Implications and recommendations

Our findings have several important implications for practice, policy, and research. The P4HP programme represents a unique initiative that places both empowerment and diet quality at the centre of prenatal nutritional care. While empowerment strategies are still relatively uncommon in nutrition interventions, despite their recognised potential [29, 31], our results demonstrate their effectiveness. The improvement in total diet quality scores, particularly for fish, iodine, and vitamin D intake, provides empirical support for empowerment-based approaches in supporting dietary changes during pregnancy.

The significant variation in effectiveness between practices (with improvements ranging up to 14 points) underscores the importance of identifying and sharing best practices among healthcare providers. Moreover, the successful collaboration between midwives and dietitians established in this study provides a valuable model for future collaborative care initiatives. As recommended in research by Beulen et al. [33], the P4HP programme adhered to key recommendations for promoting healthy dietary intake during pregnancy, including accessibility, personalised guidance, and the incorporation of dietitian consultations. These characteristics align with other successful dietary interventions that have shown small but significant improvements in pregnancy outcomes, including reduced maternal blood pressure and preterm delivery rates [93].

Future research should explore the applicability of these findings among populations with lower income levels than those in the current study, thus broadening the scope of generalizability. Additionally, assessing the cost-effectiveness of the P4HP programme would be valuable for informing broader implementation. From a public health perspective, our findings suggest that empowerment-based approaches could be beneficial in other health promotion contexts, particularly where behavior change is a primary goal. These approaches could contribute to theoretical frameworks by providing innovative practice-based evidence to support health interventions [28, 31].

## Conclusion

Pregnancy often introduces unique psychological and physiological factors that may influence dietary preferences and habits. Recognizing the role of maternal nutrition in the health outcomes of both the mother and the baby, our study evaluated the effectiveness of the P4HP programme using a C-RCT among 342 pregnant women. The observed improvement in diet quality underscores the potential of the P4HP programme as an effective intervention for improving diet quality during pregnancy. This study shows the need for support and counselling regarding weight gain during pregnancy for women of all empowerment levels and presents increased empowerment regarding gestational weight gain in the intervention group. This research lays the foundation for future investigations into the mechanisms through which empowerment can contribute to improved maternal and child health outcomes.

## Supplementary Information

Supplementary Material 1.

Supplementary Material 2.

Supplementary Material 3.

Supplementary Material 4.

## Data Availability

The datasets used and analysed during the current study are available from the corresponding author upon reasonable request.

## Abbreviations

95% CI: 95% Confidence interval
BMI: Body mass index
C-RCT: Cluster randomised controlled trial
DHD-P: The Dutch Healthy Diet index for pregnant women
IQR: Interquartile range
LOCF: Last Observation Carried Forward
MI: Motivational Interviewing
P4HP: Power 4 a Healthy Pregnancy
PRES: Pregnancy-Related Empowerment Scale
QOL: Quality of Life
SD: Standard deviation
SRH: Self-Rated Health
