# Correction to: An empowerment programme to improve diet quality during pregnancy– the Power 4 a Healthy Pregnancy cluster randomised controlled trial

**DOI:** 10.1186/s12889-025-21687-7

**Published:** 2025-02-13

**Authors:** Renske M. van Lonkhuijzen, Jeanne H. M. de Vries, Elske Brouwer‑Brolsma, Susanne Cremers, Janine P. M. Faessen, Edith J. M. Feskens, Annemarie Wagemakers

**Affiliations:** 1 Health and Society, Department of Social Sciences, Wageningen University & Research, Hollandseweg 1, Wageningen, 6706KN The Netherlands; 2 Division of Human Nutrition & Health, Wageningen University & Research, Stippeneng 4, Wageningen, 6708WE The Netherlands; 3 Bilthoven, The Netherlands

**Correction to:** ***BMC Public Health 25*** **,** ***338 (2025).***

10.1186/s12889-025-21344-z

The original publication of this article contained the following error:

- The values for the total diet quality score improvement in the abstract were incorrect, the correct values were shown in the results section.

The incorrect and correct information is listed in this correction article, this does not affect the results nor conclusions of the article. The original article has been updated.

**Incorrect**

The total diet quality score significantly improved during pregnancy in the intervention group compared to the control group (4.28; 95% CI: 7.87 to 0.70; *p* = 0.019), particularly driven by improvements in the scores for vitamin D, iodine, and fish.

**Correct**

The total diet quality score significantly improved during pregnancy in the intervention group compared to the control group (4.28; 95% CI: 1.00 to 7.56; *p* = 0.011), particularly driven by improvements in the scores for vitamin D, iodine, and fish.

