# Supplementary material for: An empowerment programme to improve diet quality during pregnancy – the Power 4 a Healthy Pregnancy cluster randomised controlled trial
Source: BMC Public Health. 2025 Jan 27;25:338. doi: 10.1186/s12889-025-21344-z (PMC11771105; doi:10.1186/s12889-025-21344-z)
Supplement: Supplementary file 1 — Supplementary Material 1. [file 12889_2025_21344_MOESM1_ESM.docx]

**Additional file 1:** **Characteristics of the midwifery practices participating in the C-RCT**

| Nr. | Phase | Group RCT | Randomization | Number of participant inclusions | Number of participants with complete dataset (no missing at all times) | Mean (SD) T0 | Mean (SD) T1 |
| --- | --- | --- | --- | --- | --- | --- | --- |
| 1^**,***^ | Pilot + RCT | Intervention | Directly into intervention group | 5+21 | 10 | 134.0 (18.1) | 138.9 (23.0) |
| 2 | Pilot | Not included |  | 3 | 2 | 144.0 (26.2) | 158.0 (8.5) |
| 3 | RCT | Control | Randomized | 29 | 15 | 133.5 (15.7) | 132.1 (18.2) |
| 4^*^ | RCT | Intervention | Randomized | 25 | 20 | 140.1 (16.1) | 144.1 (15.9) |
| 5^*^ | RCT | Control | Randomized | 26 | 14 | 140.4 (18.4) | 135.2 (19.6) |
| 6 | RCT | Intervention | Randomized | 26 | 14 | 138.4 (18.9) | 142.3 (13.7) |
| 7 | RCT | Control | Randomized | 27 | 14 | 132.3 (18.4) | 140.7 (16.3) |
| 8^**^ | RCT | Intervention | Randomized | 9 | 1 | 133.4 (14.0) | 133.0 (15.6) |
| 9 | RCT | Control | Randomized | 26 | 14 | 139.2 (15.4) | 135.5 (16.3) |
| 10 | RCT | Control | Randomized | 20 | 12 | 134.7 (19.0) | 141.7 (16.4) |
| 11^*^ | RCT | Intervention | Randomized | 32 | 18 | 126.8 (18.3) | 138.7 (15.3) |
| 12^*^ | RCT | Control | Randomized | 28 | 11 | 135.7 (17.4) | 141.3 (16.9) |
| 13 | RCT | Intervention | Randomized | 26 | 16 | 130.5 (16.4) | 138.3 (15.7) |
| 14^*^ | RCT | Intervention | Randomized | 21 | 15 | 140.0 (11.7) | 140.4 (13.3) |
| 15 | RCT | Control | Randomized | 0 | 0 | N/A | N/A |
| 16^***^ | RCT | Intervention | Directly into intervention group | 14 | 12 | 136.5 (13.8) | 147.7 (17.9) |
| 17 | RCT | Intervention | Directly into intervention group | 4 | 2 | 144.3 (12.5) | 133.0 (N/A) |
| ** located in the area of Regio Foodvalley, one of eight municipalities in the center of the Netherlands (Barneveld, Ede, Nijkerk, Rhenen, Renswoude, Scherpenzeel, Veenendaal, and Wageningen)* | | | | | | | |
| *** Midwifery practices with an existing collaboration with a dietician* | | | | | | | |
| **** First two consultations provided by the dietitian* | | | | | | | |
