# Supplementary material for: An empowerment programme to improve diet quality during pregnancy – the Power 4 a Healthy Pregnancy cluster randomised controlled trial
Source: BMC Public Health. 2025 Jan 27;25:338. doi: 10.1186/s12889-025-21344-z (PMC11771105; doi:10.1186/s12889-025-21344-z)
Supplement: Supplementary file 3 — Supplementary Material 3. [file 12889_2025_21344_MOESM3_ESM.docx]

**Additional file 3: Questionnaires for 'An empowerment program to improve diet quality during pregnancy – The Power 4 a Healthy Pregnancy cluster randomized controlled trial'**

**Questionnaire 1: sociodemographics , empowerment, and health**

| 1 | Which midwifery practice are you affiliated with? |  |
| --- | --- | --- |
| 2 | How many weeks pregnant are you now (approximately)? |  |
| 3 | What is your first and last name? |  |
| 4 | What is your phone number ? |  |
| 5 | What is your email address? |  |
| 5 | What is your year of birth? |  |
| 6 | What are the four digits of your zip code? |  |
| 7 | In which country are you born? |  |
| 8 | Do both of your parents have the same country of birth as you? | Yes, my parents were both born in the same country as me  No, my parents were not both born in the same country as me |
| 8a | In which country was your mother born? |  |
| 8b | In which country was your father born? |  |
| 9 | Do you already have children? | No, I don't have children yet  Yes, 1 child  Yes, 2 children  Yes, 3 children  Yes, 4 children  Yes, 5 children or more |
| 10 | Which training(s) have you completed? (multiple answers possible) | Secondary education  Secondary vocational education  Higher professional education  University education  Other |
| 11 | Which categories best describe your current employment situation? (multiple answers possible) | I do paid work, per week average (in hours):  I do volunteer work (unpaid)  I do the housework/I take care of my family  I receive benefits  I am on the Sickness Benefits Act/WAO  Other |
| 12 | What is your marital status? | Single  Living together  Married  Divorced  Widowed |
| 13 | What is your living situation? | I live alone  I live with my partner  I live with my partner and child(ren)  I live with my children  I live with other family members  Other |
| 14 | What is your personal net monthly income? | €1,000 or less  €1,001 to €1,500  €1,501 to €2,000  €2,001 to €2,500  €2,501 or more  I do not know |
| 15 | What is the net monthly income of your household? | €1,000 or less  €1,001 to €1,500  €1,501 to €2,000  €2,001 to €2,500  €2,501 to €3,000  €3,001 to €3,500  €3,501 to €4,000  €4,001 to €4,500  €4,501 or more  I do not know |
| 16 | What is your height in cm? |  |
| 17 | What is your current weight in kilograms? |  |
| 18 | I can ask my health care provider about my pregnancy. | Completely disagree  Somewhat disagree  Somewhat agree  Strongly agree |
| 19 | I have enough time with my health care provider to discuss my pregnancy. | Completely disagree  Somewhat disagree  Somewhat agree  Strongly agree |
| 20 | My health care provider listens to me. | Completely disagree  Somewhat disagree  Somewhat agree  Strongly agree |
| 21 | My health care provider respects me. | Completely disagree  Somewhat disagree  Somewhat agree  Strongly agree |
| 22 | I expect my health care provider to respect my decisions about my pregnancy. | Completely disagree  Somewhat disagree  Somewhat agree  Strongly agree |
| 23 | My health care provider respects my decision, even if it is different than her/his recommendation. | Completely disagree  Somewhat disagree  Somewhat agree  Strongly agree |
| 24 | I take responsibility for the decisions I make about my pregnancy like eating healthy food. | Completely disagree  Somewhat disagree  Somewhat agree  Strongly agree |
| 25 | I can tell when I have made a good health choice. | Completely disagree  Somewhat disagree  Somewhat agree  Strongly agree |
| 26 | Since I started prenatal care, I have been making more decisions about my health. | Completely disagree  Somewhat disagree  Somewhat agree  Strongly agree |
| 27 | Women need to share experiences with other women when they are pregnant. | Completely disagree  Somewhat disagree  Somewhat agree  Strongly agree |
| 28 | I share my feelings and experiences with other women. | Completely disagree  Somewhat disagree  Somewhat agree  Strongly agree |
| 29 | I know if I am gaining the right amount of weight during my pregnancy. | Completely disagree  Somewhat disagree  Somewhat agree  Strongly agree |
| 30 | I have a right to ask questions when I don't understand something about my pregnancy. | Completely disagree  Somewhat disagree  Somewhat agree  Strongly agree |
| 31 | I am able to change things in my life that are not healthy for me. | Completely disagree  Somewhat disagree  Somewhat agree  Strongly agree |
| 32 | I am doing what I can to have a healthy baby. | Completely disagree  Somewhat disagree  Somewhat agree  Strongly agree |
| 33 | If something is going wrong in my pregnancy, I know who to talk to. | Completely disagree  Somewhat disagree  Somewhat agree  Strongly agree |
| 34 | Do you usually see a solution to problems and difficulties that others see as hopeless? | Yes, usually  SometimesNo |
| 35 | Do you usually find that things that happen to you in everyday life are difficult to understand? | Yes, usually  SometimesNo |
| 36 | Do you usually find your daily life to be a source of personal satisfaction? | Yes, usually  SometimesNo |
| 37 | Overall, how do you rate your health status? | 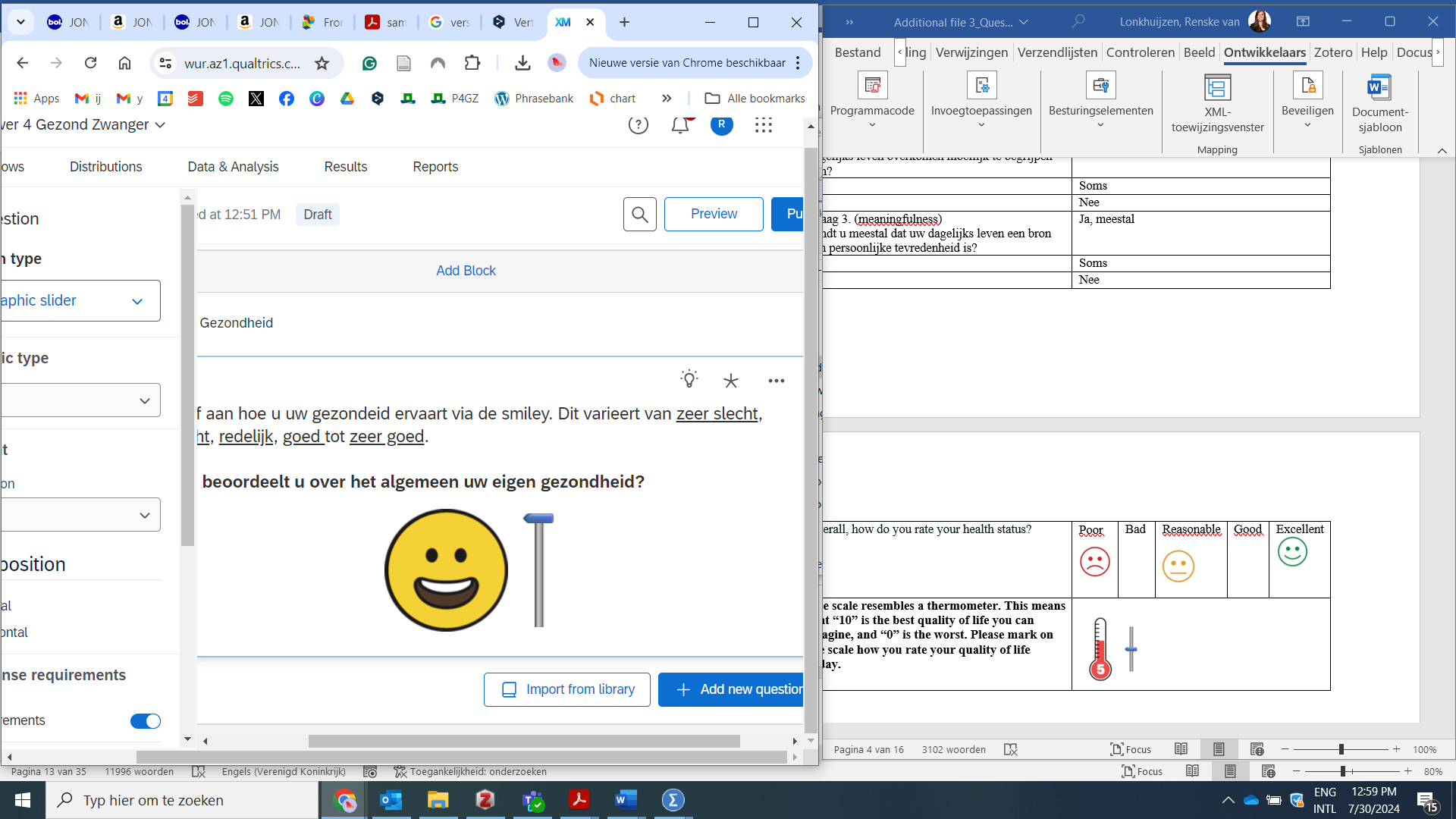 |
| 38 | The scale resembles a thermometer. This means that “10” is the best quality of life you can imagine, and “0” is the worst. Please mark on the scale how you rate your quality of life today. | 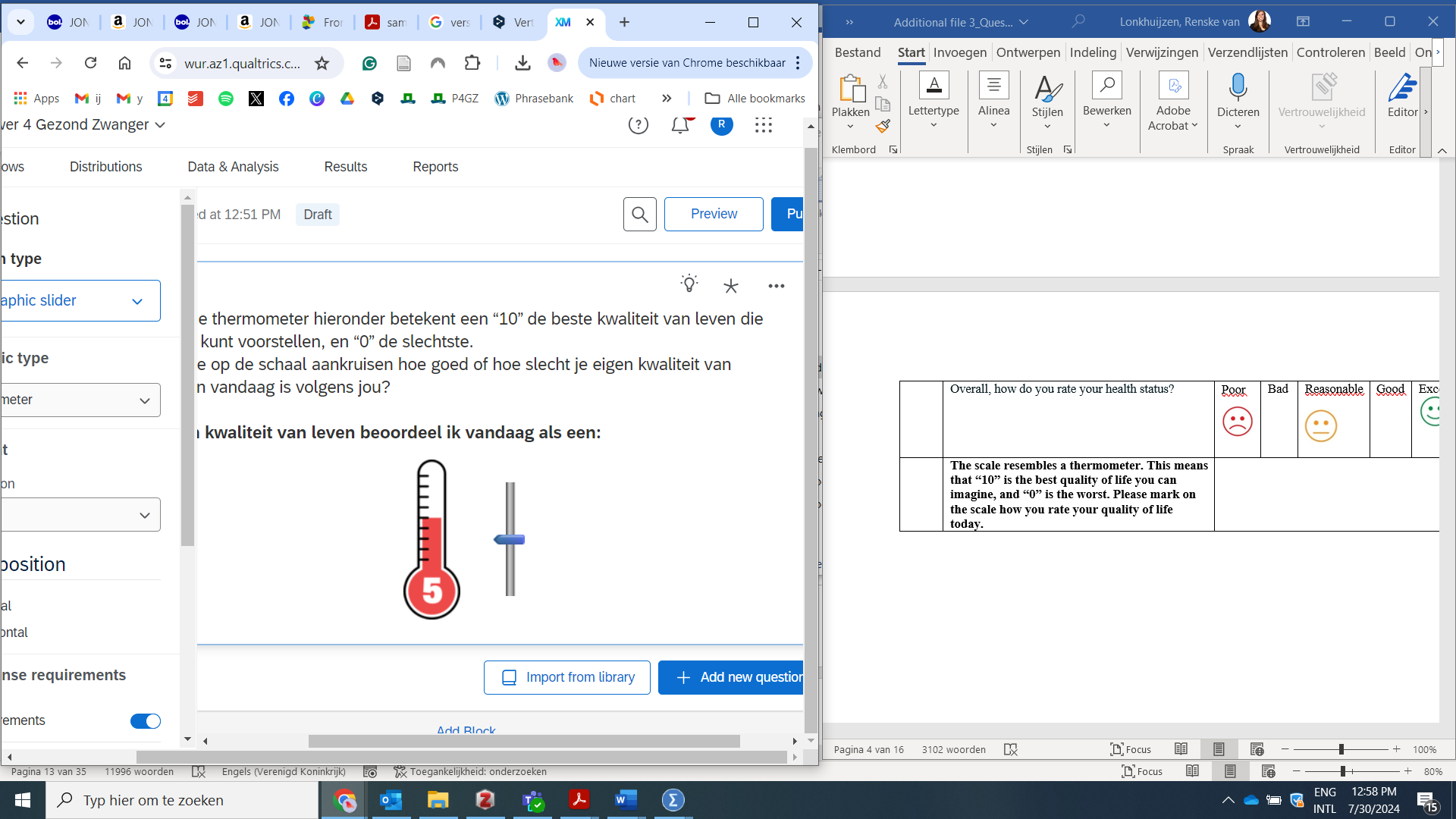 |

**Questionnaire 2: DHD-P**

| **Q** | **Question** | **Answer options** |
| --- | --- | --- |
| **1** | How many weeks pregnant are you? | Between 0 and 10 weeks |
|  |  | More than 10 weeks |
| **2a** | Do you use supplements that contain folic acid? | Yes |
|  |  | No |
| **2b** | how many micrograms ( mcg ) of folic acid are in a supplement? | less than 200 micrograms |
|  |  | 200 micrograms |
|  |  | 400 micrograms |
|  |  | 600 micrograms or more |
| **2c** | How many folic acid supplements do you take per day? | Less than 1 |
|  |  | 1 |
|  |  | 2 |
|  |  | 3 |
| **3a** | Do you use supplements that contain vitamin D? | Yes |
|  |  | No |
| **3b** | how many micrograms ( mcg ) of vitamin D are in one supplement? | less than 5 micrograms |
|  |  | 5 micrograms |
|  |  | 10 micrograms |
|  |  | 15 micrograms or more |
| **3c** | How many vitamin D supplements do you take per day? | Less than 1 |
|  |  | 1 |
|  |  | 2 |
|  |  | 3 |
| **4a** | Do you use supplements that contain iron? | Yes |
|  |  | No |
| **4b** | Approximately how many milligrams (mg) of iron are in one supplement? | 5 milligrams or less |
|  |  | 10 milligrams |
|  |  | 15 milligrams |
|  |  | 20 milligrams or more |
| **4c** | How many iron supplements do you take per day? | Less than 1 |
|  |  | 1 |
|  |  | 2 |
|  |  | 3 |
| **5** | Do you use supplements that contain vitamin A? | Yes |
|  |  | No |
| **6a** | How many days a week do you eat muesli or (breakfast) grains, such as oatmeal and corn flakes? | I don't eat muesli or (breakfast) grains |
|  |  | Less than 1 day per week |
|  |  | 1 or 2 days a week |
|  |  | 3 or 4 days a week |
|  |  | 5 or 6 days a week |
|  |  | Every day |
| **6b** | How many tablespoons ? | 1-2 tablespoons |
|  |  | 3-4 tablespoons |
|  |  | 5-6 tablespoons |
|  |  | 7-8 tablespoons |
|  |  | 9-10 tablespoons |
|  |  | 11 tablespoons or more |
| **6c** | What kind of muesli and grains do you eat? | Whole grain without sugar |
|  |  | Refined breakfast cereals |
|  |  | Both whole grain and refined breakfast cereals |
| **7** | How many croissants and/or currant buns do you eat on average per week? | No |
|  |  | Less than 1 day per week |
|  |  | 1 per week |
|  |  | 2 per week |
|  |  | 3 per week |
|  |  | 4 or more per week |
| **8a** | How much rusk, crispbread or crackers do you eat on average per day? | No |
|  |  | Less than 1 |
|  |  | 1 piece |
|  |  | 2 pieces |
|  |  | 3 pieces |
|  |  | 4 or more |
| **8b** | What kind of rusk, crispbread or crackers do you eat? | Usually brown/ whole wheat |
|  |  | Mostly white |
|  |  | Both brown/whole wheat and white |
| **9a** | How many slices of bread, rolls or buns do you eat on average per day? | No |
|  |  | 1 slice |
|  |  | 2 cuts |
|  |  | 3 cuts |
|  |  | 4 cuts |
|  |  | 5 or more cuts |
| **9b** | What types of bread, rolls or buns do you eat? | Usually brown/ whole wheat |
|  |  | Mostly white |
|  |  | Both brown/whole wheat and white |
| **10a** | Do you usually spread your bread, crispbread or rusk with low-fat margarine, margarine or butter? | No |
|  |  | Yes |
| **10b** | What do you usually spread on your bread, crispbread or rusk? | With low-fat margarine , diet low-fat margarine , diet margarine or margarine |
|  |  | With ( semi-skimmed ) butter |
|  |  | Both low-fat margarine and butter |
| **11a** | How many days a week do you eat cheese as a topping? | No |
|  |  | Less than 1 day per week |
|  |  | 1-2 days a week |
|  |  | 3-4 days a week |
|  |  | 5-6 days a week |
|  |  | Every day |
| **11b** | How many slices of bread, crispbread or rusk do you top with cheese on such a day? | 1 |
|  |  | 2 |
|  |  | 3 |
|  |  | 4 |
|  |  | 5 |
|  |  | 6 or more |
| **12a** | How many days a week do you eat processed meats as a topping? | No |
|  |  | Less than 1 day per week |
|  |  | 1-2 days a week |
|  |  | 3-4 days a week |
|  |  | 5-6 days a week |
|  |  | Every day |
| **12b** | How many slices of bread, crispbread or rusk do you top with cold cuts on such a day? | 1 |
|  |  | 2 |
|  |  | 3 |
|  |  | 4 |
|  |  | 5 |
|  |  | 6 or more |
| **13a** | How many days a week do you eat liver products as a topping? | No |
|  |  | Less than 1 day per week |
|  |  | 1 day per week |
|  |  | 2 days a week |
|  |  | 3 days a week |
|  |  | 4 or more days a week |
| **13b** | How many slices of bread, crispbread or rusk do you top with liver products, such as pâté, on such a day? | 1 |
|  |  | 2 |
|  |  | 3 |
|  |  | 4 or more |
| **14a** | How many days a week do you eat sweet toppings? | No |
|  |  | Less than 1 day per week |
|  |  | 1-2 days a week |
|  |  | 3-4 days a week |
|  |  | 5-6 days a week |
|  |  | Every day |
| **14b** | How many slices of bread, crispbread or rusk do you cover with sweet toppings on such a day? | 1 |
|  |  | 2 |
|  |  | 3 |
|  |  | 4 |
|  |  | 5 |
|  |  | 6 or more |
| **15a** | How many days a week do you drink milk, buttermilk or other dairy drinks without sugar? | No |
|  |  | Less than 1 day per week |
|  |  | 1-2 days a week |
|  |  | 3-4 days a week |
|  |  | 5-6 days a week |
|  |  | Every day |
| **15b** | How many glasses/cups of milk, buttermilk or dairy drinks without sugar do you drink on such a day? | 1 |
|  |  | 2 |
|  |  | 3 |
|  |  | 4 |
|  |  | 5 |
|  |  | 6 or more |
| **16a** | How many days a week do you drink chocolate milk or other dairy drinks with sugar? | No |
|  |  | Less than 1 day per week |
|  |  | 1-2 days a week |
|  |  | 3-4 days a week |
|  |  | 5-6 days a week |
|  |  | Every day |
| **16b** | How many glasses/cups of chocolate milk or other dairy drinks with sugar do you drink on such a day? | 1 |
|  |  | 2 |
|  |  | 3 |
|  |  | 4 |
|  |  | 5 |
|  |  | 6 or more |
| **17a** | How many days a week do you eat dairy products such as custard, pudding, (fruit) yoghurt, (fruit) cottage cheese or ice cream? | No |
|  |  | Less than 1 day per week |
|  |  | 1-2 days a week |
|  |  | 3-4 days a week |
|  |  | 5-6 days a week |
|  |  | Every day |
| **17b** | How many bowls of these dairy products do you eat on such a day? | Less than 1 bowl |
|  |  | 1 bowl |
|  |  | 2 bowls |
|  |  | 3 bowls |
|  |  | 4 bowls |
|  |  | 5 or more |
| **18a** | How many days a week do you use soy drink or soy yogurt? | No |
|  |  | Less than 1 day per week |
|  |  | 1-2 days a week |
|  |  | 3-4 days a week |
|  |  | 5-6 days a week |
|  |  | Every day |
| **18b** | How many glasses/bowls of soy drink/yogurt do you use on such a day? | Less than 1 |
|  |  | 1 |
|  |  | 2 |
|  |  | 3 |
|  |  | 4 or more |
| **19** | How many plates or cups of soup do you eat on average per week? | No |
|  |  | Less than 1 day per week |
|  |  | 1-2 per week |
|  |  | 3-4 per week |
|  |  | 5-6 per week |
|  |  | 7 or more |
| **20a** | How often have you eaten legumes in the past month? | No |
|  |  | Once a month​ |
|  |  | 2 times a month |
|  |  | 3 times a month |
|  |  | Once a week |
|  |  | More than once a week |
| **20b** | How many serving spoons of legumes (±60 grams) do you eat? | 1 serving spoon |
|  |  | 2 serving spoons |
|  |  | 3 serving spoons |
|  |  | 4 serving spoons |
|  |  | 5 serving spoons |
|  |  | 6 or more serving spoons |
| **21a** | How many days a week do you eat boiled or stir-fried vegetables? | No |
|  |  | Less than 1 day per week |
|  |  | 1-2 days a week |
|  |  | 3-4 days a week |
|  |  | 5-6 days a week |
|  |  | Every day |
| **21b** | How many serving spoons of vegetables (±50 grams) do you eat on such a day? | 1 serving spoon |
|  |  | 2 serving spoons |
|  |  | 3 serving spoons |
|  |  | 4 serving spoons |
|  |  | 5 serving spoons |
|  |  | 6 or more serving spoons |
| **22a** | How many days a week do you eat raw vegetables? | No |
|  |  | Less than 1 day per week |
|  |  | 1-2 days a week |
|  |  | 3-4 days a week |
|  |  | 5-6 days a week |
|  |  | Every day |
| **22b** | How many bowls of raw vegetables (of ±50 grams) do you eat on such a day? | Less than 1 bowl |
|  |  | 1 bowl |
|  |  | 2 bowls |
|  |  | 3 bowls |
|  |  | 4 bowls |
|  |  | 5 or more bowls |
| **23a** | How many days a week do you eat pasta? | No |
|  |  | Less than 1 day per week |
|  |  | 1-2 days a week |
|  |  | 3-4 days a week |
|  |  | 5-6 days a week |
|  |  | Every day |
| **23b** | How many serving spoons of pasta (±50 grams) do you eat on such a day? | 1 serving spoon |
|  |  | 2 serving spoons |
|  |  | 3 serving spoons |
|  |  | 4 serving spoons |
|  |  | 5 serving spoons |
|  |  | 6 or more |
| **23c** | What type of pasta do you eat? | Usually whole wheat pasta |
|  |  | Usually white pasta |
|  |  | Both whole wheat and white pasta |
| **24a** | How many days a week do you eat rice? | No |
|  |  | Less than 1 day per week |
|  |  | 1-2 days a week |
|  |  | 3-4 days a week |
|  |  | 5-6 days a week |
|  |  | Every day |
| **24b** | How many serving spoons of rice (±50 grams) do you eat on such a day? | 1 serving spoon |
|  |  | 2 serving spoons |
|  |  | 3 serving spoons |
|  |  | 4 serving spoons |
|  |  | 5 serving spoons |
|  |  | 6 or more |
| **24c** | What type of rice do you usually eat? | Usually whole wheat rice |
|  |  | Mostly white rice |
|  |  | Both whole wheat and white rice |
| **25a1** | How many days a week do you eat red meat? | No |
|  |  | Less than 1 day per week |
|  |  | 1-2 days a week |
|  |  | 3-4 days a week |
|  |  | 5-6 days a week |
|  |  | Every day |
| **25a2** | How many days a week do you eat processed meat? | No |
|  |  | Less than 1 day per week |
|  |  | 1-2 days a week |
|  |  | 3-4 days a week |
|  |  | 5-6 days a week |
|  |  | Every day |
| **25a3** | How many days a week do you eat chicken and other poultry? | No |
|  |  | Less than 1 day per week |
|  |  | 1-2 days a week |
|  |  | 3-4 days a week |
|  |  | 5-6 days a week |
|  |  | Every day |
| **25b** | How many portions do you eat on such a day? | Less than 1 |
|  |  | 1 |
|  |  | 1 ½ |
|  |  | 2 |
|  |  | 2 ½ |
|  |  | 3 or more |
| **26** | eat liver? | Yes |
|  |  | No |
| **27a** | How many days a week do you eat soy products with a hot meal? | No |
|  |  | Less than 1 day per week |
|  |  | 1-2 days a week |
|  |  | 3-4 days a week |
|  |  | 5-6 days a week |
|  |  | Every day |
| **27b** | How many portions of soy products do you eat on such a day? | 1 |
|  |  | 2 |
|  |  | 3 |
|  |  | 4 or more |
| **28a** | How many days a week do you eat savory snacks? | No |
|  |  | Less than 1 day per week |
|  |  | 1-2 days a week |
|  |  | 3-4 days a week |
|  |  | 5-6 days a week |
|  |  | Every day |
| **28b** | How many portions do you eat on such a day? | Less than 1 |
|  |  | 1 |
|  |  | 1 ½ |
|  |  | 2 |
|  |  | 2 ½ |
|  |  | 3 or more |
| **29** | How often did you eat a portion of lean fish in the past month? | No |
|  |  | Less than once a month |
|  |  | Once a month​ |
|  |  | 2 times a month |
|  |  | 3 times a month |
|  |  | 4 times a month or more |
| **30** | How often did you eat a portion of oily fish in the past month? | No |
|  |  | Less than once a month |
|  |  | Once a month​ |
|  |  | 2 times a month |
|  |  | 3 times a month |
|  |  | 4 times a month or more |
| **31a** | Do you usually use fat for the preparation of hot meals? | No |
|  |  | Yes |
| **31b** | What type(s) of fat do you usually use for the preparation of hot meals? NB. Multiple answers possible . | Butter |
|  |  | Margarine or baking product from a packet |
|  |  | Margarine or baking product out An bottle |
|  |  | Oil |
| **32a** | How many days a week do you eat sauces such as mayonnaise, garlic sauce or ketchup with a hot meal or with savory snacks? | No |
|  |  | less than 1 day per week |
|  |  | 1-2 days a week |
|  |  | 3-4 days a week |
|  |  | 5-6 days a week |
|  |  | Every day |
| **32b** | How many tablespoons of sauce do you eat on such a day? | 1 tablespoon |
|  |  | 2 tablespoons |
|  |  | 3 tablespoons |
|  |  | 4 tablespoons |
|  |  | 5 tablespoons |
|  |  | 6 or more |
| **33a** | How many days a week do you eat cheese with a hot meal or in between? | No |
|  |  | Less than 1 day per week |
|  |  | 1-2 days a week |
|  |  | 3-4 days a week |
|  |  | 5-6 days a week |
|  |  | Every day |
| **33b** | How many portions of cheese do you eat on such a day? | 1-2 servings |
|  |  | 3-4 servings |
|  |  | 5-6 servings |
|  |  | 7-8 servings |
|  |  | 9-10servings |
|  |  | 11 or more |
| **34a** | How many days do you eat peanuts or nuts? | No |
|  |  | Less than 1 day per week |
|  |  | 1-2 days a week |
|  |  | 3-4 days a week |
|  |  | 5-6 days a week |
|  |  | Every day |
| **34b** | How many handfuls of peanuts or nuts (of ±25 grams) do you eat on such a day? | 1 handful |
|  |  | 2 hands |
|  |  | 3 hands |
|  |  | 4 hands |
|  |  | 5 hands |
|  |  | 6 or more |
| **35a** | How many days a week do you eat chips or snacks? | No |
|  |  | Less than 1 day per week |
|  |  | 1-2 days a week |
|  |  | 3-4 days a week |
|  |  | 5-6 days a week |
|  |  | Every day |
| **35b** | How many handfuls of chips or snacks do you eat on such a day? | 1-2 handfuls |
|  |  | 3-4 handfuls |
|  |  | 5-6 handfuls |
|  |  | 7-8 hands |
|  |  | 9-10 hands |
|  |  | 11 or more |
| **36** | How many large cookies, cakes or pies do you eat on average per week? | No |
|  |  | Less than 1 per week |
|  |  | 1 per week |
|  |  | 2 per week |
|  |  | 3 per week |
|  |  | 4 or more per week |
| **37a** | How many days a week do you eat small cookies or gingerbread? | No |
|  |  | Less than 1 day per week |
|  |  | 1-2 days a week |
|  |  | 3-4 days a week |
|  |  | 5-6 days a week |
|  |  | Every day |
| **37b** | How many small cookies or slices of gingerbread do you eat on such a day? | 1-2 pieces |
|  |  | 3-4 pieces |
|  |  | 5-6 pieces |
|  |  | 7-8 pieces |
|  |  | 9-10 pieces |
|  |  | 11 or more |
| **38a** | How many days a week do you eat chocolate or chocolates? | No |
|  |  | Less than 1 day per week |
|  |  | 1-2 days a week |
|  |  | 3-4 days a week |
|  |  | 5-6 days a week |
|  |  | Every day |
| **38b** | How many blocks of chocolate or chocolates do you eat on such a day? | 1-2 pieces |
|  |  | 3-4 pieces |
|  |  | 5-6 pieces |
|  |  | 7-8 pieces |
|  |  | 9-10 pieces |
|  |  | 11 or more |
| **39a** | How many days a week do you eat fruit? | No |
|  |  | Less than 1 day per week |
|  |  | 1-2 days a week |
|  |  | 3-4 days a week |
|  |  | 5-6 days a week |
|  |  | Every day |
| **39b** | How many portions/pieces of fruit do you eat on such a day? | Less than 1 |
|  |  | 1 |
|  |  | Sometimes 1 sometimes 2 |
|  |  | 2 or more |
| **40a** | How many days a week do you drink fruit juice? | No |
|  |  | Less than 1 day per week |
|  |  | 1-2 days a week |
|  |  | 3-4 days a week |
|  |  | 5-6 days a week |
|  |  | Every day |
| **40b** | How many glasses of fruit juice do you drink on such a day? | 1 glass |
|  |  | 2 glasses |
|  |  | 3 glasses |
|  |  | 4 glasses |
|  |  | 5 glasses |
|  |  | 6 glasses or more |
| **41a** | How many days a week do you drink soft drinks, fruit lemonade, sports drinks or energy drinks ? | No |
|  |  | Less than 1 day per week |
|  |  | 1-2 days a week |
|  |  | 3-4 days a week |
|  |  | 5-6 days a week |
|  |  | Every day |
| **41b** | How many glasses of soft drink do you drink on such a day? | 1 glass |
|  |  | 2 glasses |
|  |  | 3 glasses |
|  |  | 4 glasses |
|  |  | 5 glasses |
|  |  | 6 glasses or more |
| **42a** | How many days a week do you drink green or black tea? | No |
|  |  | Less than 1 day per week |
|  |  | 1-2 days a week |
|  |  | 3-4 days a week |
|  |  | 5-6 days a week |
|  |  | Every day |
| **42b** | How many glasses/cups/cups of green or black tea do you drink on such a day? | 1 cup |
|  |  | 2 cups |
|  |  | 3 cups |
|  |  | 4 cups |
|  |  | 5 cups |
|  |  | 6 or more cups |
| **43** | Add sugar or honey to the tea | Virtually always |
|  |  | Sometimes |
|  |  | Never |
| **44a** | How many cups/cups of coffee do you drink on average per day? | No |
|  |  | 1 cup |
|  |  | 2 cups |
|  |  | 3 cups |
|  |  | 4 cups |
|  |  | 5 or more cups |
| **44b** | What type of coffee do you usually drink? NB. Multiple answers possible . | Coffee made with a paper filter or percolator, coffee pods or instant coffee |
|  |  | Coffee from the machine, coffee from cups |
|  |  | Coffee made with a cafetiere , cooking coffee, Greek or Turkish coffee |
|  |  | I know not |
| **45** | Do you put sugar in your coffee? | Virtually always |
|  |  | Sometimes |
|  |  | Never |
| **46** | drink alcohol? | Yes |
|  |  | No |
| **47** | Is salt added to potatoes, vegetables, meat, rice or pasta during the preparation of your hot meal? | Virtually always |
|  |  | Sometimes |
|  |  | Never |
| **48** | Do you add salt, maggi or ketjap to your meal or dish at the table? | Virtually always |
|  |  | Sometimes |
|  |  | Never |
