# Supplementary material for: An empowerment programme to improve diet quality during pregnancy – the Power 4 a Healthy Pregnancy cluster randomised controlled trial
Source: BMC Public Health. 2025 Jan 27;25:338. doi: 10.1186/s12889-025-21344-z (PMC11771105; doi:10.1186/s12889-025-21344-z)
Supplement: Supplementary file 4 — Supplementary Material 4. [file 12889_2025_21344_MOESM4_ESM.docx]

| Component | | DHD<=meanT1 (n=107) | DHD>meanT1 (n=102) | Difference between groups |
| --- | --- | --- | --- | --- |
| Total score T1 | | 136.31 | 163.68 | 27.37 |
| 1 | Vegetables | 5.05 | 7.71 | 2.66 |
| 2 | Fruit | 6.93 | 8.94 | 2.01 |
| 3 | Whole-grain products | 7.05 | 8.19 | 1.14 |
| 4 | Legumes | 4.47 | 8.27 | 3.8 |
| 5 | Nuts | 2.57 | 6.43 | 3.86 |
| 6 | Dairy products | 4.99 | 4.77 | -0.22 |
| 7 | Fish | 3.34 | 5.15 | 1.81 |
| 8 | Caffeine | 7.38 | 8.43 | 1.05 |
| 9 | Fat and oils | 5.46 | 7.45 | 1.99 |
| 10 | Coffee | 7.43 | 8.63 | 1.2 |
| 11 | Red meat | 9.75 | 9.91 | 0.16 |
| 12 | Processed meat | 4.89 | 6.85 | 1.96 |
| 13 | Sugar-containing beverages | 6.37 | 7.99 | 1.62 |
| 14 | Alcohol | 10 | 10 | 0 |
| 15 | Salt | 8.7 | 8.78 | 0.08 |
| 16 | Unhealthy choices | 2 | 3.7 | 1.7 |
| 17 | Vitamin D | 6.92 | 8.36 | 1.44 |
| 18 | Vitamin A | 8.55 | 8.96 | 0.41 |
| 19 | Soy | 9.91 | 9.8 | -0.11 |
| 20 | Iodine | 4.61 | 5.34 | 0.73 |

**Additional file 4: Diet quality scores of groups that score lower and higher on average diet quality at T1, without distinction between intervention and control group.**
